# Cardiovascular exercise and burden of arrhythmia in patients with atrial fibrillation - A randomized controlled trial

**DOI:** 10.1371/journal.pone.0170060

**Published:** 2017-02-23

**Authors:** Ane Katrine Skielboe, Thomas Quaade Bandholm, Stine Hakmann, Malene Mourier, Thomas Kallemose, Ulrik Dixen

**Affiliations:** 1 Department of Cardiology, Copenhagen University Hospital Amager & Hvidovre, Hvidovre, Denmark; 2 Clinical Research Centre, Copenhagen University Hospital Amager & Hvidovre, Hvidovre, Denmark; 3 Physical Medicine and Rehabilitation Research – Copenhagen (PMR-C), Department of Physical Therapy, Copenhagen University Hospital Amager & Hvidovre, Hvidovre, Denmark; 4 Department of Orthopedic Surgery, Copenhagen University Hospital Amager & Hvidovre, Hvidovre, Denmark; Universita degli Studi Magna Graecia di Catanzaro, ITALY

## Abstract

**Background:**

Physical activity at moderate-high intensity is recommended to prevent lifestyle diseases. Patients with atrial fibrillation are at risk of a sedentary lifestyle due to fear of exercise-induced episodes of atrial fibrillation. The burden of arrhythmia can be reduced by physical exercise. The effect of exercise intensity on burden of atrial fibrillation needs to be studied further.

**Methods and results:**

In a 12-week randomized controlled trial, 76 patients with paroxysmal/persistent atrial fibrillation were allocated to perform exercise at either low intensity or high intensity (50% and 80% of maximal perceived exertion, respectively). Primary outcome was burden of AF measured by daily electrocardiography-reporting during 12 weeks. Secondarily, change in maximal oxygen uptake (peak VO_2_) and 1-year hospitalization was compared between low and high intensity exercise. Sixty-three patients completed the follow-up. In the intention-to-treat analysis, we found no statistical difference in burden of atrial fibrillation between low and high intensity exercise (incidence rate ratio 0.742, 95% CI 0.29–1.91, P = 0.538). No serious adverse events were reported and there was no difference in hospitalization between the two exercise groups. Both exercise groups improved significantly in peak VO_2_ (low intensity: 3.62 mL O_2_/kg/min, SD 3.77; high intensity: 2.87 mL O_2_/kg/min, SD 4.98), with no statistical difference between-groups (mean difference: 0.76 mL O_2_/kg/min, 95% CI -3.22–1.7).

**Conclusions:**

High intensity physical exercise was not superior to low intensity physical exercise in reducing burden of atrial fibrillation. HI exercise was well tolerated; no evidence of an increased risk was found for HI compared to LI exercise. Larger studies are required to further prove our findings.

**Trial registration:**

ClinicalTrials.gov NCT01817998

## 1. Introduction

Atrial fibrillation (AF) is the most common cardiac rhythm disturbance in the Western World, affecting ~2% of the general population [1]. With improved survival from other cardiovascular disease and longer lifespan, the incidence is expected to increase even more [2]. The arrhythmia has great impact on daily life, causing a more sedentary lifestyle due to symptoms or fear of provoking episodes of AF [3]. A sedentary lifestyle increases the risk of cardiovascular disease, hypertension, diabetes, and obesity [4]. These conditions are all risk factors for AF, and can possibly perpetuate AF [5–8].

In most observational studies of healthy individuals, both a sedentary lifestyle and endurance exercise are associated with increased risk of incident AF [9–11]. A recent systematic review regarding elite exercise and AF risk states a higher risk of AF among elite athletes [12], although others argue that this is overestimated [13]. In the latest meta-analysis concerning health benefits of physical exercise for patients with all types of AF, exercise led to improvements in frequency control, lower risk of AF-relapse after ablation, and increased quality of life [14].

Awareness has increased of using physical activity as supplementary treatment to patients with paroxysmal/persistent atrial fibrillation (PAF). Recently, high intensity (HI) cardiovascular exercise has been shown to reduce the burden of AF compared to a passive control group [15]. It remains unknown, if the positive effect of cardiovascular exercise on the burden of AF is related to exercise intensity. The aim of the study was to investigate if 12 weeks of HI cardiovascular exercise would reduce the burden of AF more than low intensity (LI) cardiovascular exercise in patients with PAF.

## 2. Methods

### 2.1 Study design

The study was an assessor-blinded, randomized, controlled, superiority trial, in which patients were assigned 1:1 to a 12-week program of physical exercise at LI or HI. Assessments were performed before first exercise session (baseline) and after 12 weeks (follow-up). Throughout week 4–16, patients transmitted an electrocardiography (ECG) twice daily and in case of AF symptoms.

The reporting of the study follows the Consolidated Reporting of Reporting of Trials (CONSORT), using the extension for non-pharmacological trials [16] (S1 CONSORT Checklist), and the reporting of the exercise intervention follows the Template for Intervention Description and Replication checklist (TIDieR) [17]. The present study was developed in accordance with the Declaration of Helsinki and has approval from the National Committee on Health Research Ethics (H-2-2012-048). All patients gave their written informed consent, and the trial was registered at ClinicalTrials.Gov, March 2013 (NCT01817998), https://clinicaltrials.gov/ct2/show/NCT01817998

### 2.2 Participants

Patients were included, using consecutive sampling, from The Department of Cardiology at Copenhagen University Hospital, Amager and Hvidovre, in the time period 2013–15. Possible eligible patients were found by screening lists of admissions from the cardiology ward and the cardiology out-patient clinics at the two hospitals. Eligibility criteria were admission with International Classification of Disease (ICD)-10 codes Z035A (suspected arrhythmia), I489 (atrial fibrillation or flutter unspecified), R002 (palpitations), Z035 (suspected cardiovascular disease), I471 (supraventricular tachycardia with narrow QRS-interval), R008A (cardiac arrhythmia unspecified), and I480 (paroxysmal atrial fibrillation). Medical journals were reviewed according to inclusion and exclusion criteria, and eligible patients offered participation by mail or telephone.

Inclusion criteria were paroxysmal or persistent AF (PAF) documented on ECG, age≥18 years, and written informed consent. Exclusion criteria were permanent AF, substantial language barrier, severe health conditions making physical exercise impossible, life expectancy shorter than one year, or signs of cardiac disease during baseline tests.

### 2.3 Randomization

#### 2.3.1. Sequence generation

After stratification for sex, patients were randomly assigned 1:1 to either LI or HI exercise using a simple shuffling envelopes procedure. All patients were assigned with a unique identification number. Each identification number was assigned randomly with the letter A or B, by drawing from an opaque envelope. Until the first exercise session, a note revealing which intensity each letter represented was kept in a sealed envelope. The physiotherapists conducting the exercise sessions were allocated to a group before the randomization.

#### 2.3.2. Allocation concealment and implementation

To ensure concealment, randomization was generated by an employee at the hospital with no connection to the trial. The physiotherapists received the envelope revealing the exercise intensity at the first session.

### 2.4 Blinding

All assessors and analysts were blinded to the intervention allocation. Patients were blinded to the study hypothesis, and they were not informed of what intervention they were receiving, but it is highly possible that they were able to figure it out. They were informed not to reveal any detail about their exercise group when performing follow-up tests, to preserve blinding of the research team. All data was registered in an anonymous database by the patients’ identification number.

### 2.5 Intervention

Over 12 weeks, supervised exercise sessions of 60 minutes each were conducted twice weekly at The Department of Physiotherapy, Copenhagen University Hospital, Amager and Hvidovre. To ensure close supervision of the patients during exercise, the participants were subdivided into small groups of ten patients exercising with one physiotherapist. Exercise was planned by experienced cardiac rehabilitation physiotherapists.

Intensity was measured on the Borg scale (Rating of Perceived Exertion 1–20) [18] to adjust for individual physical improvement and to avoid using heart rate as measuring scale. Borg-20 scale is valid for measuring exercise intensity and ensures standardization of the exercise program so each individual exercise at the same relative intensity [18,19]. Assessing exercise intensity with heart rate could be inaccurate in patients receiving medical frequency control or in case a patient had AF during exercise. LI exercise was defined as 50% of maximum perceived exertion (Borg 11–13) and HI as 80% of maximum perceived exertion (Borg 16–18). Each exercise session consisted of 10 min warm up (Borg 7–10), 20 min interval exercising on ergometer bike (with allocated intensity), 20 min varying circuit exercise on the floor (with allocated intensity), and 10 min cool down. Exercise intensity progressed gradually over the first five weeks; during week 1 the patients got familiar with using the Borg scale for adjustment of exercise intensity, and during week 2–5 biking, walking, and circuit exercises were introduced, and exercise intensity increased weekly until reaching the scheduled 50% and 80% of maximum, respectively. Intensity remained constant during week 6–12, and volume increased substantially. The complete exercise program is specified in S1 Appendix A.

### 2.6 Outcomes

At baseline and follow-up, blinded assessments were performed by trained medical personnel. Patients had an advanced echocardiography, a 24-hour measurement of blood pressure and heart rate, cardiopulmonary exercise test (CPET) for measuring maximal oxygen uptake (peak VO_2_) and a questionnaire of previous medical history.

#### 2.6.1 Descriptives

Demographic variables were age (years), sex, ethnicity (white or other), height (cm), BMI (kg/m^2^), smoking (yes, no, former), and alcohol (men: ≤14 or >14 units of alcohol weekly, women: ≤ 7 or > 7 units of alcohol weekly). Medical history was obtained by self-reported questionnaire and verified in medical records; type of AF (paroxysmal, persistent), time since diagnosis of AF (months), hypertension, diabetes, ischemic heart disease, metabolic disease, chronic obstructive pulmonary disease, heart failure, and daily cardiac medication. Left ventricular ejection fraction (LVEF, %), left atrial volume index (LA volume, mL/m^2^), and left ventricular internal diameter end diastolic (LViDd, cm) were measured by echocardiography. Significant heart valve disease and pulmonary hypertension were noted if present. Blood pressure (mmHg) and heart rate (bpm) measured for 24 hours were presented as mean systolic and diastolic values during day time and night time. Overall exercise participation was assessed as percentage participation.

Echocardiography was performed by a cardiologist to rule out structural cardiac disease, using Philips iE33, Philips Medical System 6.0.2.144. LA volume and LVEF were assessed using the modified Simpson’s method, estimating the average volumes from apical two- and four- chamber views.

Assessment of blood pressure and heart rate was performed by laboratory technicians using ScottCare ABP 320, CardioView Dx 4.0.8, and Rozinn RZ153+, software edition v10.6, respectively. Echocardiography was analyzed by a cardiologist in accordance with European standard guidelines [20].

#### 2.6.2 Primary outcome

The primary efficacy outcome was burden of AF in the time period from week 4 to week 16 of exercise. It was calculated as the ratio of ECGs with AF and total number of ECGs (ECG_AF_/ECG_total_).

ECG was measured using a patient-triggered handheld ECG-device (Zenicor^®^), software edition 3.0. Diagnosis with AF was defined as minimum 10 s of coherent irregular rhythm and lack of a defined p-wave ahead of each QRS-complex. Analysis was performed independently twice by a physician and evaluated by a blinded cardiologist in case of questionable diagnosis. ECGs not possible to diagnose unambiguously were excluded from analysis. Sensitivity (96%) and specificity (92%) regarding AF has been determined previously [21].

#### 2.6.3 Secondary outcomes

The secondary outcomes were change in peak VO_2_ and overall hospital admissions.

Change in peak VO_2_ was measured from baseline to follow-up (week 12) to assess the effect on physical fitness of LI and HI exercise, respectively. CPETs were performed on ergometer bike protocolled with three min warm up followed by stepwise increase in load. Two trained assistants executed the test and analyzed ECG-monitoring and blood pressure continuously. The test was considered valid at complete exhaustion with respiratory exchange rate (ReR) ≥1.0. In case of any adverse events during CPET, the test was interrupted instantly. Maximal exercise capacity was termed peak VO_2_ as recommended when testing non-healthy subjects [22].

Peak VO_2_ (mL O_2_/min/kg) was measured with breath-by-breath gas analysis using CosMed K4 b2^®^. Calibration procedure was performed as specified in the manual in reference to humidity, ambient temperature, and barometric pressure; room air calibration was performed before each test, delay and gas calibration daily, volume calibration weekly. Raw data was processed by running 8-breath averaging (mean) and peak VO_2_ determined as the maximum value within the last 60 s of the test [23].

Hospital admissions were assessed from baseline (week 1) to one year after last exercise session. Electronic medical records were reviewed twice by two independent reviewers for admissions caused by recurrent AF verified on ECG, heart failure, stroke, radio frequency ablation, surveillance for medical antiarrhythmic treatment, or pacemaker implantation. Death of any cause was reported.

All patients were evaluated for adverse events during the intervention period; if a patient felt unwell during exercise, a blinded physician examined the patient and reported the event. This included chest pain, dizziness, suspected recurrence of AF, and musculoskeletal injuries.

### 2.7 Statistics

Prior to the analyses, data were entered blinded and validated by double entry. All analyses were performed blinded and according to a pre-established analysis plan. Any deviation from this plan is accounted for below (please see Deviations from the trial registration). Statistical analyses were conducted using the STATA statistical software package version 13.1.

#### 2.7.1 Descriptives

Baseline data were compared between intervention groups, HI and LI. For demographics, Pearson χ^2^-test or Fisher’s exact test was performed for categorical variables, independent t-test or Wilcoxon rank-sum test was performed depending on distribution. Normally distributed data were presented with mean and standard deviation (SD), non-parametric data with median and inter quartile range (IQR).

#### 2.7.2 Primary outcome

Initially, Poisson regression was performed to analyze the difference in AF burden between exercise groups, but goodness of fit-test on deviance was poor (P<0.0001). Under assumption of over-dispersed data, negative binomial regression analysis was performed and confirmed (dispersion parameter = 2.7). The likelihood-ratio test strongly suggested dispersion parameter≠0, confirming that a negative binomial regression model was a more appropriate fit. Results were reported as incidence rate ratio (IRR) for HI exercise compared to LI exercise with 95% confidence intervals (95% CI). P-value of less than 0.05 was considered statistically significant for all primary and secondary analyses.

Before un-blinding data, six possible confounding variables were identified; age, gender, LA volume, mean heart rate (24 hours), baseline peak VO_2_, and treatment with beta-blocker. Each variable was evaluated by individual extraction from the full regression model, and confounding variables were kept in the adjusted regression analysis (LA volume, mean heart rate and treatment with beta-blocker medication).

Both intention-to-treat (ITT) and per-protocol (PP) analyses were performed. All patients who were randomized according to the opened envelopes were included in the ITT analysis, whereas patients participating in 2/3 (65%) of the exercise sessions were included in the PP analysis.

Dose-response relationship between participation and AF burden was tested, as well as effect modification by participation.

#### 2.7.3 Secondary outcomes

Independent t-test was used to analyze change in peak VO_2_. Two analyses were performed; one restricted to patients with ReR ≥1.0 in combination with sinus rhythm on ECG during the CPETs, and one analysis containing all participants. Hospital admissions were analyzed with Fisher’s Exact Test or χ^2^-test when appropriate.

### 2.8 Deviations from the trial registration

Physical capacity was measured as peak VO_2_ instead of Six Minute Walk Test as described in the protocol (S1 Clinical Trial Protocol). We got access to the equipment after designing the study before start up and changed it due to the improved quality it added to the study.

The duration of each exercise session was 60 minutes instead of alternately 60 and 90 minutes for logistical reasons.

The study was registered at Clinical Trials three months after the first patient was included; unfortunately we were unaware of the great importance of pre-registration. However, no changes were made to the study during these three months.

Before analyzing data, it became clear that some patients transmitted several ECGs daily without marking it as a symptomatic episode. To reduce possible information bias, burden of AF was assessed additionally as number of days with AF on minimum one ECG. ITT analysis was performed under the same conditions as the primary analysis of AF-ratio and likewise reported as IRR with 95% CI.

To detect any time-dependent difference in first AF-episode after the exercise sessions started, Cox proportional regression analysis was performed and reported as hazard ratios (HR) with 95% CI for HI exercise compared to LI exercise.

Baseline data from cardiac ultrasound and 24-hour measurement of blood pressure/heart rate is used for description of the study population. Follow-up data are part of more comprehensive analyses to be reported later, as well as quality of life. Blood samples were collected and are stored in a freezer for future analyses. Reports on these data will hold a clear reference to the primary trial and its trial identifier.

### 2.9 Sample size

An average rate of burden AF-episodes in patients with PAF is not known, and is difficult to estimate. We assumed the LI group would have approximately 20 ECGs with AF during the time of monitoring (week 4–16). A mean reduction in AF burden of 25% was considered the least clinically relevant. Enrolling 60 patients would make it possible to detect this difference and achieve a power of 90% for two-sided α = 0.05 (95% CI). Our sample size estimation was based on an independent two-sample t-test. Taking into account an attrition rate of 20%, a minimum of 72 patients needed to be included.

## 3. Results

### 3.1 Participant flow

**Fig 1:**
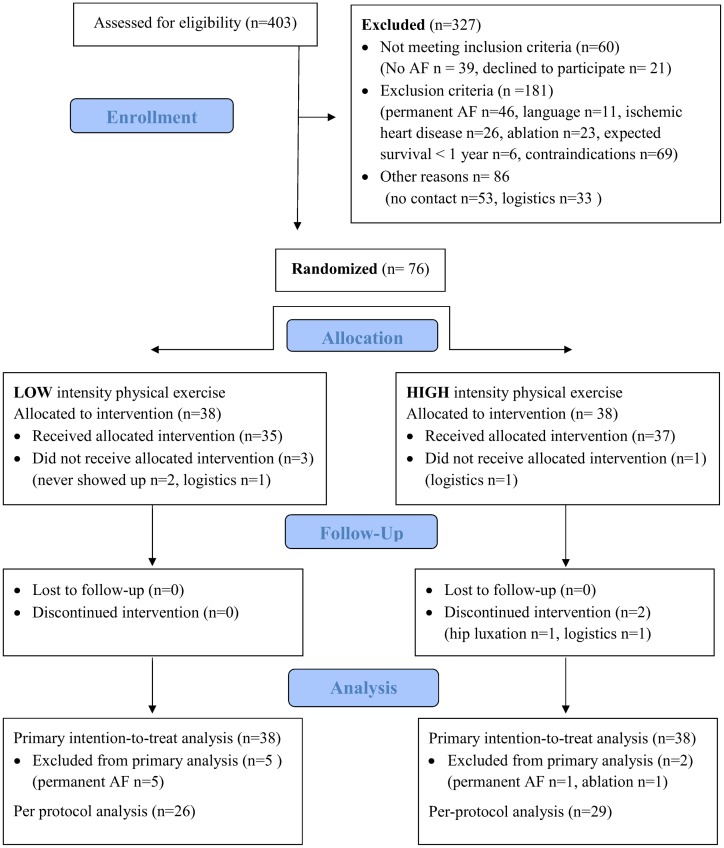
Patient flow diagram for low and high intensity physical exercise.

In the primary screening of participants, 403 patients were found potentially eligible. Of these, 277 patients did not meet inclusion criteria after reviewing medical records. The remaining 126 patients were asked to participate; 50 patients declined mainly due to employment or logistics (Fig 1). Overall, 76 patients were included, of these, 63 patients completed the full 12-week exercise intervention. After randomization, seven patients were excluded for various reasons; six patients were screening failures with permanent AF, and one patient was excluded from analysis due to cardiac ablation. During the 12-week intervention time, six patients dropped out; four patients did not receive intervention, of these, two patients never showed up (allocated to LI exercise) and two patients had to withdraw their consent due to logistics (one from each exercise group); two participants allocated to HI exercise discontinued intervention, one due to concomitant disease and one due to logistics.

### 3.2 Recruitment

The study was carried out from 1^st^ January 2013- 28^th^ February 2015.

### 3.3 Baseline data

All variables, except treatment with vitamin K-antagonist, were equally distributed between intervention groups (Table 1).

**Table 1:** Baseline characteristics of low and high intensity exercise groups.

Variable	Level	Low intensity (N = 33)	High intensity (N = 37)
Gender	Male	19 (57.6%)	22 (59.4%)
Race	White	32 (97.0%)	36 (97.3%)
Age, years		63.8 (3.3)	61.4 (3)
Height, cm		177 (9)	178 (11)
BMI, kg/m^2^		30.1 (9.2)	29.1 (7.9)
Peak VO_2_, mL O_2_/kg/min		23.0 (9.7)	21.0 (6.9)
Tobacco	Former	17 (51.5%)	18 (48.6%)
	no	13 (39.4%)	12 (32.4%)
	yes	3 (9.1%)	7 (18.9%)
Alcohol, National	≤	30 (90.9%)	32 (86.5%)
Recommendations^a^	>	3 (9.1%)	5 (13.5%)
Habitual exercise, hours/week	<1 h	16 (48.5%)	10 (27.0%)
	1–3 h	13 (39.4%)	19 (51.4%)
	3–6 h	3 (9.1%)	4 (10.8%)
	>6 h	1 (3.0%)	4 (10.8%)
Type AF	paroxysmal	18 (54.5%)	16 (43.2%)
	persistent	15 (45.5%)	21 (56.8%)
Hypertension	yes	16 (48.5%)	21 (56.8%)
Diabetes mellitus	yes	4 (12.1%)	6 (16.2%)
Ischemic heart disease	yes	2 (6.1%)	2 (5.4%)
Metabolic disease	yes	3 (9.1%)	4 (10.8%)
COPD	yes	1 (3.0%)	3 (8.1%)
Heart failure,	yes	3 (9.1%)	0 (0%)
Medication			
*β-blocker*	yes	22 (66.7%)	23 (62.2%)
*Ca*^*2+*^*-blocker*	yes	8 (24.2%)	11 (29.7%)
*ACE+ARB*^b^	yes	9 (27.3%)	14 (37.8%)
*Digoxin*	yes	2 (6.1%)	4 (10.8%)
*1c anti arrhythmica*	yes	5 (15.2%)	11 (29.7%)
*Novel Oral Anticoagulants*	yes	13 (39.4%)	11 (29.7%)
*Vitamin K Antagonists*	yes	4 (12.1%)	17 (45.9%)
*Acetylsalicylic Acid*	yes	3 (9.1%)	7 (18.9%)
*Statins*	yes	14 (42.4%)	18 (48.6%)
*Diuretics*	yes	7 (21.2%)	5 (13.5%)
LVEF	<50%	4 (12.9%)	5 (14.3%)
	50–59%	18 (58.1%)	17 (48.6%)
	>60%	11 (35.5%)	13 (37.1%)
LA volume^c^, mL/m^2^		36.55 (29.3–43.9)	37.8 (28.2–42.5)
LViDd, cm		5.01 (0.58)	4.97 (0.61)
AF duration, months^c^		33.6 (10.9–62.2)	20.6 (7.7–54.3)
Heart rate, bpm		66 (10)	67 (12)
Blood pressure^c^, mmHg	Day	125 (12) / 74 (8)	128 (13) / 73 (14)
	Night	118 (16) / 66 (10)	118 (19) / 68 (10)
Participation^c^, %		87 (70–92)	87 (65–92)
ECG total^c^, n		161 (23–169)	164 (29–178)

Data presented as mean (SD) and number (%) if not otherwise stated

^a^Recommended intake in Denmark: Women ≤7 units/week, men ≤15 units/week

^b^ACE = Angiotensin Converting Enzyme Inhibitor, ARB = Angiotensin-II Receptor Antagonist

^c^Reported as median (IQR)

### 3.4 Outcomes

The reporting of ECGs was high; a total of 9,971 ECGs were transmitted from the population of these 4,586 ECGs from LI exercise (98.3% being interpretable) and 5,385 ECGs from HI exercise (98.9% being interpretable). Total number of AF episodes in the interpretable ECG’s were n = 553 and n = 534 for LI and HI exercise, respectively. On average, each participant reported median 1.9 ECGs/day (IQR 1.5–2.0) and 2 ECGs/day (IQR 1.5–2.1) for LI and HI physical exercise, respectively.

#### 3.4.1 Primary analysis for the primary outcome (ITT)

**Fig 2:**
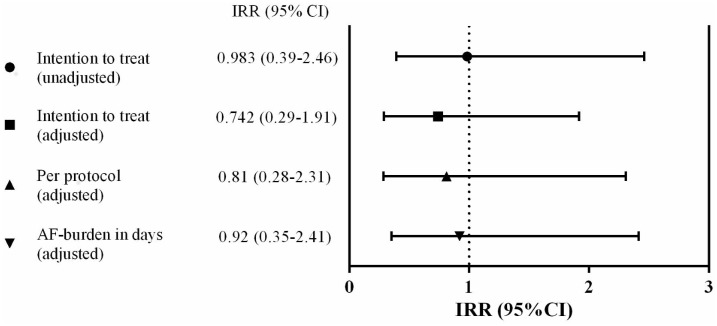
Incidence Rate Ratio (IRR) for burden of atrial fibrillation, high compared to low intensity exercise. IRR<1 favors high intensity exercise.

In the unadjusted regression analysis, the burden of AF was not statistically different between the exercise groups (IRR 0.983, 95% CI 0.39–2.46, P = 0.971), Fig 2. Similarly, after adjusting for predefined confounders (LA volume, use of beta blocker, and baseline heart rate), the IRR decreased, but the effect of HI exercise remained not statistically different from LI exercise (IRR 0.742, 95% CI 0.29–1.91, P = 0.538).

Three patients differed considerably from the rest by having AF more than 95% of the time, a condition very close to permanent AF. Excluding them from the analysis did not change the overall outcome, IRR 0.89 (95% CI 0.37–2.14, P = 0.799). Participation did not modify the outcome, IRR = 1.0 (95% CI 0.95–1.06, P = 0.442), and we found no dose-response relationship between participation and AF burden, IRR = 1.02 (95% CI 0.99–1.05, P = 0.324).

#### 3.4.2 Secondary analysis for the primary outcome (PP)

In total, 52 patients fulfilled the protocol with a minimum participation rate of 65% of all exercise sessions. Similar to the ITT-analysis, the burden of AF was not statistically different between the exercise groups in the adjusted PP analysis (IRR 0.81, 95% CI 0.28–2.31, P = 0.416), Fig 2.

#### 3.4.3 Secondary analysis for the secondary outcomes

In the analysis of peak VO_2_, two CPETs were excluded due to unrecognized disease during the test (pneumonia and erysipelas, respectively). When considering CPET with ReR>1.0 and normal ECG, both groups improved their peak VO_2_ significantly during the 12-week exercise, mean (LI) 3.62 mL O_2_/kg/min (SD 3.77), and mean (HI) 2.87 mL O_2_/kg/min (SD 4.98). We found no significant difference in improvement of peak VO_2_ between the exercise groups (mean diff. -0.76 mL O_2_/kg/min, 95% CI -3.22–1.70). Likewise, when including all CPET’s (mean diff. 0.07 mL O_2_/kg/min, 95% CI -2.24–2.38).

During follow-up, 38 admissions were registered, Table 2. There was no difference in overall hospitalization for LI exercise compared to HI exercise. Admissions with recurrence of AF were most frequent, but with no significant difference between LI and HI exercise, P = 0.465.

**Table 2:** Hospital admissions during follow-up for LI and HI physical exercise.

	Low intensity	High intensity	
Follow-up time, days	448.5 (18.4)	426.6 (101.1)	
Recurrent AF	17 (89.5%)	13 (68.4%)	P = 0.465^a^
Ablation	0 (0%)	5 (26.3%)	
Antiarrhythmic medical treatment	2 (10.5%)	0 (0%)	
Heart failure, n (%)	0 (0%)	1 (5.3%)	
Pacemaker implantation	0 (0%)	0 (0%)	
Stroke	0 (0%)	0 (0%)	
Total	19 (100%)	19 (100%)	

Data presented as mean (SD) and number (%)

^a^χ^2^-test of admissions with recurrent AF for LI compared to HI exercise groups

#### 3.4.4 Ancillary analyses

Analyzing AF burden as number of days with AF, showed no significant reduction in AF burden between HI and LI exercise (IRR 0.92, 95% CI 0.35–2.41, P = 0.865), Fig 2.

We found no significant difference in time to first AF episode; patients exercising at HI did not have their first AF episode later than patients exercising at LI (HR = 1.1, 95% CI 0.6–1.9, P = 0.806).

### 3.5 Adverse events

An adverse event was defined as any reported physical inconvenience during the exercise sessions. In case the patients had symptoms that could be related to AF or otherwise needed medical evaluation, they were examined by a blinded physician. If confirmed and considered of significance in relation to the physical exercise it was reported as a major adverse event. In total, nine patients were medically examined during the trial. No serious adverse events related to the exercise sessions were registered. One patient paused exercise sessions shortly due to increasing symptoms of arrhythmia, which in the later analysis turned out to be accumulation of benign supraventricular ectopic activity (HI-group). Two patients were admitted to the hospital after an exercise session due to AF recurrence; both episodes had started ahead of the session and were not considered to be induced by the exercise (HI-group). Three patients had AF ahead of an exercise session; two converted spontaneously (LI-group) and one had adjusted the medical treatment (HI-group). Three patients had minor complaints proven to be non-cardiac: one patient (HI-group) felt unspecific chest pain (chronic gastric reflux); one patient felt dizzy (HI-group); one patient had temporary worsening of arthritis (LI-group).

## 4. Discussion

### 4.1 Principal findings

In this randomized controlled trial, we studied the effect of cardiovascular exercise on burden of AF among patients with paroxysmal or persistent AF. Over 12 weeks, 76 patients participated in a program of either LI or HI physical exercise and assessed their heart rhythm daily. The main finding was that HI exercise was not superior to LI exercise in reducing the burden of AF during the exercise intervention period.

So far, most interventional exercise trials have included patients with permanent AF or patients undergoing cardiac radiofrequency ablation. Cardiovascular exercise has been shown to improve resting heart rate and to some extend reduce the need for medical treatment in these patients [24–27]. After radiofrequency ablation, exercise as part of lifestyle changes has improved the long-term maintenance of sinus rhythm [28]. One study has focused on daily AF burden among patients with PAF measured by seven-day Holter monitoring and the AF severity scale questionnaire [29]. The study was observational and found an association between improved physical fitness and reduction in AF symptoms and arrhythmia on ECG.

The causal pathway between improved physical fitness and the cardiac impact of exercise is not fully elucidated but includes changes in the autonomic regulation and remodeling of the atrial wall. The balance between parasympathetic and sympathetic regulation of the heart is important for preventing new episodes of AF [30,31]. While athletes often experience AF episodes triggered by a strong parasympathetic tone (e.g. in the relaxation phase after exertion), most other AF patients are under influence of a relative high sympathetic tone, which makes the atrium more susceptible to AF [30,32]. Regular physical exercise on a non-elite level reverses the autonomic balance towards a stronger parasympathetic cardiac tone, and this effect is suggested to improve rhythm regulation and prevention of AF episodes [24–28].

In our study, we could not confirm any clear difference in burden of AF between the two exercise groups. The daily reporting of ECGs was good (median 1.9–2 ECGs/day), but the average number of AF episodes was well below the expected (LI 50p = 3, IQR 0–14; HI 50p = 4, IQR 0–17). Of 63 analyzed patients, twenty patients had no AF at all (32%), so any difference became difficult to identify. It is possible the patients in general were healthier than expected due to some degree of selection bias that can occur in a clinical trial, especially with the possibility of being allocated to HI physical exercise. Moreover, using intermittent thumb ECG (Zenicor^®^) increases the risk of underestimating AF burden compared to continuous monitoring. Thumb ECG is easy to use for the patients, convenient and without any risks. Compared with 24-hour Holter monitoring, intermittent thumb ECG recording during four weeks has been proven more effective at detecting AF [33]. It could possibly discourage too many patients to complete the trial if Holter monitoring had to be used for a longer period due to the inconvenience of wearing it. The randomization procedure should ensure that a possible underestimation affects the two exercise groups equally.

A recent published trial by Malmo et al. found that AF burden was reduced after 12 weeks of high-intensity cardiovascular exercise compared to passive (*do as usual*) controls [15]. The study differed some from our study by including highly symptomatic patients and by comparing the HI exercise-intervention with passive controls. The exercise volume was slightly larger and AF burden was measured continuously by a loop recorder.

Our trial focused on studying the effect of different exercise intensities. Physical exercise prevents other lifestyle diseases in a dose-response manner [34], but based on the increased incidence of AF among elite athletes, it has been uncertain whether the same was applicable in preventing r AF. In our study, we could not significantly show that HI exercise was superior to LI exercise in reducing AF burden. Most likely, the difference in effect of LI and HI physical exercise on AF burden is smaller than expected. If exercise was maintained beyond 12 weeks, a beneficial effect on AF burden could possibly be more pronounced due to slow onset of a favorable cardiac risk profile, weight loss, and further increased exercise capacity [7]. Another explanation for our findings could be that the difference in exercise effect on AF burden does not show because the patients achieved the same physiological effect of the exercise intervention (peak VO_2_). However, peak VO_2_ was only assessed as a secondary validation of the exercise effect and any firm conclusions on this relationship should be made with caution since we do not know the exact relationship between change in VO_2_ and AF.

Both groups improved their physical fitness significantly to a level comparable with patients from other cardiac rehabilitation programs (3–4 mL O_2_/min/kg) [35]. It was expected they would improve more than patients with heart failure and ischemic heart disease. To our surprise, we did not find that HI exercise improved the physical fitness more than LI exercise did. Several factors can explain this outcome. The study was designed with a LI group, exercising at 50% of maximum perceived exertion, where only little improvement in fitness was expected. For the participants, we issued no restrictions on their daily physical activity to make it comparable to clinical practice. During the study, we experienced behavioral change among the patients, e.g. going by bike instead of by car, because they felt more confident about exercising. Moreover, it is possible that AF patients do not obtain the same additional effect on peak VO_2_ from the higher intensity. In the trial by Malmo et al., the effect of HI exercise on improvement in peak VO_2_ was smaller than expected, 3.2 mL O_2_/min/kg (SD 2.5) [15]. We find it possible that exercise has greater effect on rhythm control, even at lower intensities of exercise, than on the improvement in physical fitness. As also discussed in the paper by Malmo et al. [15], medical rhythm control and recurrent AF episodes can possibly modify the physical performance and reduce the expected additional effect seen in healthy individuals.

It was only possible to obtain partial blinding of the patients, and in some cases it was a challenge to restrict the intensity to 50% for patients at the LI exercise. Patients interested in physical exercise are more likely to volunteer to an exercise study and might be biased about the beneficial effect of exercise. We tried to prevent this effect by carefully informing both patients and the physiotherapists that we did not know which exercise intensity was most favorable for AF patients.

We performed two analyses of peak VO_2_; one analysis restricted to patients free of AF during the test and with a cut-off point at ReR≥1.00, and one analysis containing all tests. When analyzing all CPETs together, the mean improvement in VO_2_ declined, possibly reflecting inclusion of submaximal CPETs.

It is widely discussed which method is the best for assessing maximal physical fitness in patients, since ReR is affected by high age, obesity, sedentary life style, and cardiac disease [22,36]. Atwood et al. performed maximal exercise testing in 50 AF patients and achieved mean ReR = 1.06 [37]. Until recently, patients with PAF was expected to have a normal exercise capacity in sinus rhythm (if no underlying cardiac disease) [14,37], but new evidence suggests that AF patients can have reduced diastolic cardiac function thereby affecting maximal exercise capacity [35]. In lack of specific guidelines for exercise testing in AF-patients, we relied on recommendations for cardiac rehabilitation exercise testing [22], and excluded the tests from the analyses if AF was present.

Besides a tangible symptom relief, it is of consideration whether a smaller AF burden can reduce the risk of serious complications as thromboembolic events or cardiomyopathy induced by tachycardia. Observational studies suggest a higher risk of thromboembolic events in patients with a high burden of AF [38–41], and directly in relation to prolonged AF episodes [42]. We know that physical activity is associated with decreased mortality and cardiovascular disease, and it would be intriguing to find out, if a reduction in AF burden could reduce the risk of thromboembolic events additionally.

This study report important experiences to be used in future interventional studies;. for further large scale studies, measuring both rhythm on ECG as well as symptoms on the AF severity scale questionnaire will be preferable. Several patients in this study expressed that their AF episodes appeared less intense and of shorter duration, but we were not able to quantify that information, given our focus on the objective measured AF burden.

In the daily clinic, advising patients with PAF about exercise and possible limitations has been based primarily on observational studies and personal experience. It is a very heterogeneous patient group, and there are no formalized rehabilitation programs to offer. Our data showed that the patients tolerated HI exercise well, without excess adverse events or hospital admissionsand we do not find that the fear of worsening the arrhythmia should be an obstacle to performing HI exercise for preventing further lifestyle diseases. However, we recognize the need for confirming this in larger scale studies.

### 4.2 Strengths and limitations

This trial is the first randomized controlled trial in a clinical setting studying the effect of different cardiovascular exercise-intensities on burden of AF among patients with PAF. Assessment of our primary outcome burden of AF was very detailed with daily measuring of the cardiac rhythm and a high reporting rate. Both exercise participation and adherence to the study were high.

Exercise was supervised by experienced physiotherapists, who trained both LI and HI, thereby neutralizing the effects of any unequal expertise. Supervised exercise conducted at the hospital eliminates the insecurity related to self-reported home exercise and increases adherence. All participants received an intervention to equalize the positive effect of being in a trial. The vast majority of the exercise tests were performed by the same technical assistants to reduce difference in individual performance. No serious adverse events were related to the exercise intervention.

The main limitation of our study is the low burden of AF and small sample size giving no opportunity of making statistical significant conclusion. The unexpected improvement in physical fitness for LI exercise illustrates the difficulties in administering the right dose of exercise. Exercise testing is sensitive to psychological barriers and motivation, and there can be a learning effect for the patient over time.

## 5. Conclusion

In this randomized controlled trial, 12 weeks of HI exercise was not superior to LI exercise in reducing the primary outcome, burden of AF, among patients with PAF. HI exercise was well tolerated; no evidence of an increased risk was found for HI compared to LI exercise. To maintain a healthy lifestyle, AF patients should perform physical exercise, however based on our findings it is not possible to provide any specific recommendations for AF patients and further largescale studies with longer follow-up are needed.

## Supporting information

S1 CONSORT Checklist (DOCX)

S1 Appendix A Detailed 12-week physical exercise program. (DOCX)

S1 Data sheets (XLSX)

S1 Clinical Trial Protocol English Version (DOCX)

S1 Clinical Trial Protocol Original Danish Version (DOCX)
