# Supplementary material for: Cardiovascular exercise and burden of arrhythmia in patients with atrial fibrillation - A randomized controlled trial
Source: PLoS One. 2017 Feb 23;12(2):e0170060. doi: 10.1371/journal.pone.0170060 (PMC5322948; doi:10.1371/journal.pone.0170060)
Supplement: S1 Appendix A — (DOCX) [file pone.0170060.s002.docx]

# Appendix A: Detailed 12-week physical exercise program

Low intensity (LI) physical exercise was defined as 50% of maximum perceived exertion (Borg scale 11-13). High intensity (HI) physical exercise was defined as 80% of maximum perceived exertion (Borg scale 16-18). Exercise intensity started at Borg 10-13 and progressed gradually over the first five weeks (Table A.1).

During week 1, the patients got familiar with using the equipment and Borg scale for adjustment of exercise intensity. During week 2-5 biking, walking and circuit exercises on the floor were introduced (Figure A.1) and exercise intensity increased weekly until reaching the scheduled 50% or 80% of maximum, respectively, for LI and HI exercise. During week 6-12, intensity remained constant and volume increased substantially by increasing the time in active intervals and shortening the recovery time between intervals (Table A.1).

#
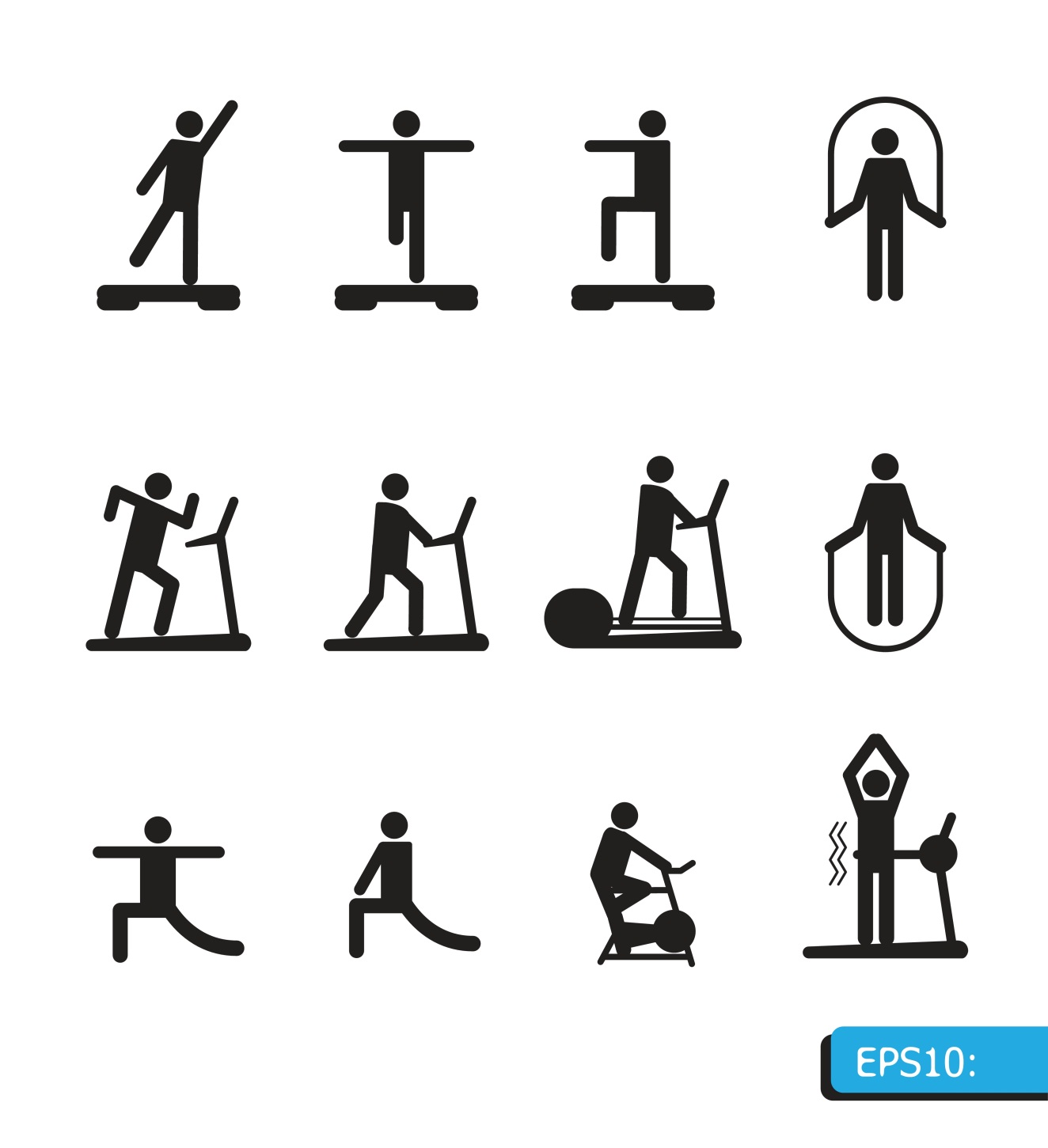
Timeline for exercise sessions, 60 minutes.

1. Warm up 5-10 min at Borg 7-10 on ergometer bike

2. Interval on ergometer, 20 min bike with allocated intensity (Table 1)

3. Interval exercises on the floor, 20 min with allocated intensity (Table 1, Fig. 1)

4. Cool down and stretch out 5-10 min

## Table A.1, Exercise intervals and perceived exertion

| Week | Ergometer bike  (active/recovery), | BORG-score | |  | Floor exercises (active/recovery), | BORG-score | |  |
| --- | --- | --- | --- | --- | --- | --- | --- | --- |
|  | min | LI | HI | | min | LI | HI | |
|  |  |  |  | |  |  |  | |
| 1-2 | 8 x (1.5/1.0) | 10-13 | 10-13 | | 8 x (1.5/1.0) | 10-13 | 10-13 | |
| 3 | 7 x (2.0/1.0) | 11-13 | 14-15 | | 7 x (2.0/1.0) | 11-13 | 14-15 | |
| 4 | 6 x (2.5/1.5) | 11-13 | 16-17 | | 6 x (2.5/1.5) | 11-13 | 14-15 | |
| 5 | 4 x (3.0/1.5) | 11-13 | 16-17 | | 4 x (3.0/1.5) | 11-13 | 14-15 | |
| 6 | 5 x (3.0/1.0) | 11-13 | 16-17 | | 5 x (3.0/1.0) | 11-13 | 14-15 | |
| 7 | 4 x (3.5/1.5) | 11-13 | 16-17 | | 4 x (3.5/1.5) | 11-13 | 16-17 | |
| 8 | 5 x (3.5/1.0) | 11-13 | 16-17 | | 5 x (3.5/1.0) | 11-13 | 16-17 | |
| 9 | 3 x (4.0/2.0) | 11-13 | 17-18 | | 3 x (4.0/2.0) | 11-13 | 16-17 | |
| 10 | 4 x (4.0/1.0) | 11-13 | 17-18 | | 8 x (1.0/1.0) | 11-13 | 16-17 | |
| 11 | 10 x (1.0/1.0) | 11-13 | 17-18 | | 4 x (4.0/1.0) | 11-13 | 16-17 | |
| 12 | 11 x (1.0/0.5) | 11-13 | 14-18 | | 5 x (4.0/1.0) | 11-13 | 16-17 | |

.

## Figure A.1: Examples of exercise intervals on the floor for LI and HI exercise:


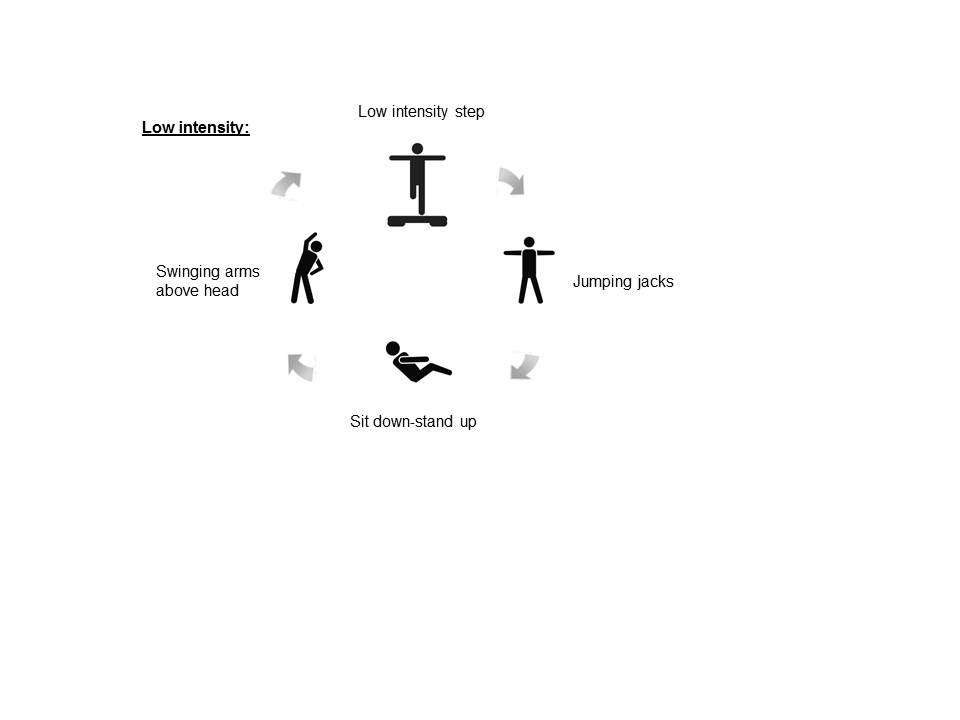


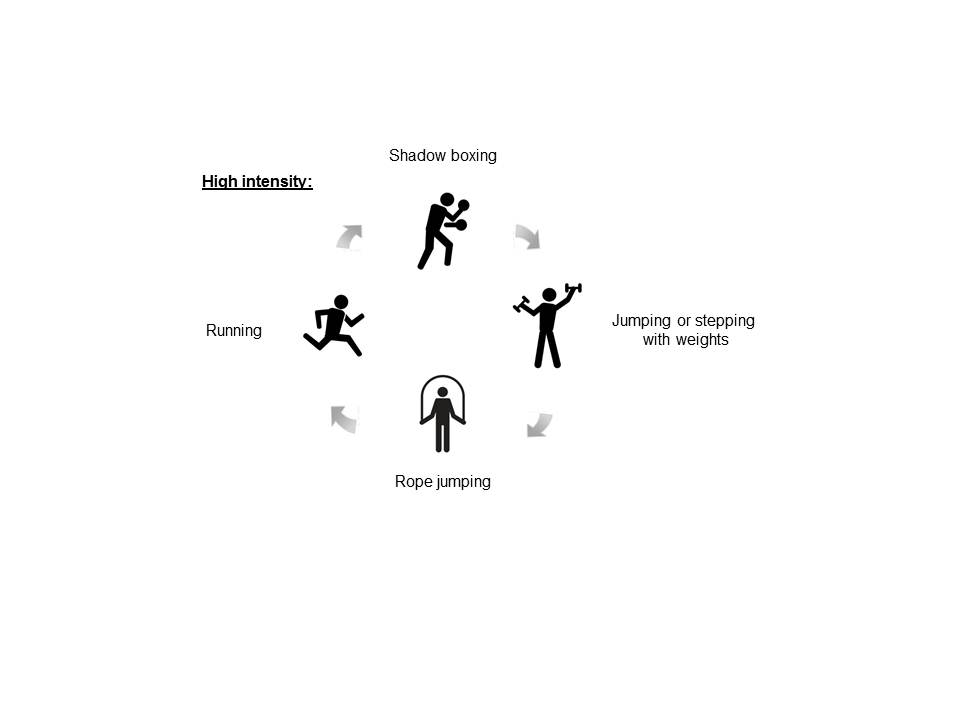


Photo: Colourbox
