# Supplementary material for: Cardiovascular exercise and burden of arrhythmia in patients with atrial fibrillation - A randomized controlled trial
Source: PLoS One. 2017 Feb 23;12(2):e0170060. doi: 10.1371/journal.pone.0170060 (PMC5322948; doi:10.1371/journal.pone.0170060)
Supplement: S1 Clinical Trial Protocol English Version — (DOCX) [file pone.0170060.s004.docx]

Protocol

Physical exercise and atrial fibrillation – a multidisciplinary interventional study

# Introduction

Atrial fibrillation (AF) is the new cardiac epidemic in the Western countries[1–3]. It can be a fatal cardiac disease especially due to the risk of stroke where 70% of the patients will be either disabled or die[4,5]. Consequences are huge both for each patient individually and for the health care economy[6].

While the incidence of ischemic heart disease has decreased, the incidence of AF has increased and is expected to increase even further, primarily due to improved treatment of other cardiac diseases and a higher life expectancy[7,8]. Besides a higher risk of hospital admissions due to AF, many patients have symptoms from AF on daily basis and feel anxious to participate in physical exercise. This affects their normal social behavior and impairs the quality of life[9].

Medical treatment is complex and can be hazardous due to side effects. Several studies of antiarrhythmic treatment have been interrupted due to severe side effects, the latest in 2011[10]. Therefore, medical treatment still requires individual assessment and close monitoring of the patient. Radio frequency ablation is only possible for a minority of AF patients and is associated with a risk of complications[11].

So far, we only have limited knowledge about the effect of physical exercise on AF. Long-term endurance exercise increases the risk of AF in time due to dilatation of the cardiac chambers[12,13]. However, moderate physical exercise prevents premature death and other life style diseases associated with an increased risk of AF (coronary artery disease, obesity, hypertension)[14–19]. The effect of physical exercise on early stages of AF is unknown and many patients are afraid of provoking episodes of AF or cardiac arrest when exercising.

Previously, our research team and a Norwegian research team have studied the effect of physical exercise in patients with permanent AF and found an improvement in exercise capacity and quality of life[20,21]. Based on these experiences, we now want to study the effect of physical exercise in an early stage of the disease.

The pathophysiological mechanisms for AF are also unclear. Studies of cardiac tissue samples show possible cardiac fibrosis in patients with AF. It is difficult though, to conduct these invasive studies, but it is possible in blood samples to assess markers of fibrosis reflecting atrial fibrosis[22–24]. Therefore, we have conducted a pilot study of the association between markers of fibrosis and AF. Our study showed that stromal derived factor (SDF)-1α, which recruits stem cells from the bone marrow to the heart, was significantly increased in patients with permanent AF compared to patients with paroxysmal AF. Moreover, the concentration of clusterin (a marker of biological aging) was increased in patients with AF compared to healthy controls. Cardiac fibrosis, concomitant heart failure and biological aging affects the risk of developing permanent AF and complications as stroke or death[25,26].

We also study the association between telomere length and AF. Telomere length and telomerase activity are specific genetic markers. Telomeres shorten in time and lose the ability of cell division, but telomerase can delay this process. A short telomere length is associated with coronary artery disease and heart failure, but so far no studies have been conducted regarding AF. In this trial, we study the effect of physical exercise on telomere length and telomerase activity, and whether short telomere length predicts the effect of physical exercise.

We expect to assess several outcomes due to the presence of AF in many clinical areas. The study is designed as a randomized interventional trial including patients with paroxysmal or persistent AF documented on an electrocardiogram. Patients will be allocated randomly to either low or high intensity physical exercise. In this design with two active arms we want to prevent a passive control group from exercising without our knowledge.

We hope to show that physical exercise can be used additionally to the regular medical treatment of arrhythmia.

# Study objectives

1. effect of physical exercise on risk of AF measured on daily electrocardiogram (Zenicor®) during the exercise intervention
2. effect of physical exercise on hospital admissions during one year after the intervention
3. effect of physical exercise on quality of life measured before and after the exercise intervention (SF36 and EHRA-classification)
4. effects of physical exercise on physical capacity measured by 6 Minute Walk Test and on ergometer bike
5. effect of physical exercise on the cardiac work load measured on echocardiography, 24-hour heart rate and rhythm, and blood pressure
6. the predictive value of telomere length, telomerase activity, copeptin and fibrosis markers on exercise effect

# Hypotheses

1. high intensity physical exercise reduces burden of AF by 20% (number of AF-episodes, time to first episode)
2. high intensity physical exercise reduces hospital admissions by 20%
3. high intensity physical exercise improves quality of life on EHRA-classification and by 20% in minimum five out of eight parameters on SF36
4. high intensity physical exercise increases maximal physical capacity by 20%
5. high intensity physical exercise improves the cardiac work load by reducing the mean heart rate and blood pressure, and improving the diastolic function.
6. a significant association between telomere length and effect of exercise as well as increased telomerase activity after the exercise intervention.

# Methods

The study is an assessor blinded, randomized controlled trial. Patients are randomized by simple drawing. For logistic reasons, an exercise team consists of ten patients. The letters A and B are equally distributed in 20 envelopes, and represents the physical exercise intensity. The sealed envelopes are all put into a larger envelope from where the patients will draw one envelope at randomization.


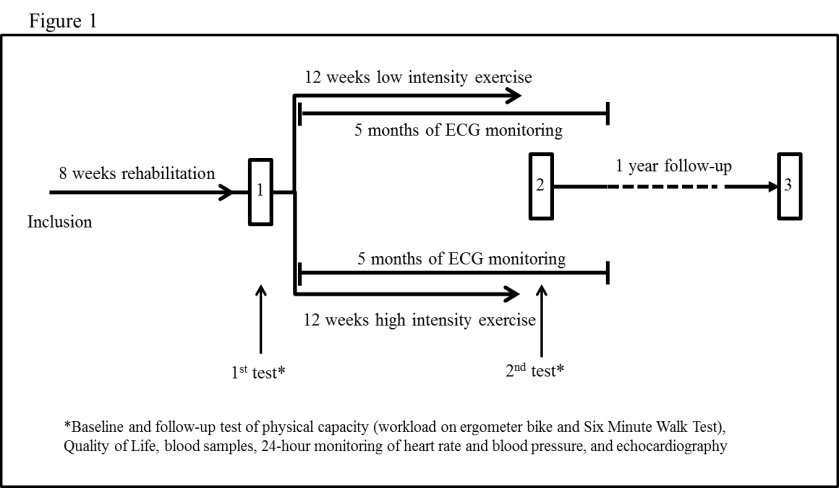
A total of 60 patients with paroxysmal/persistent AF will be included, using consecutive sampling, from the Department of Cardiology, Copenhagen University Hospital Hvidovre. After inclusion, baseline tests will be performed as follows: physical capacity measured on Six Minute Walk Test and maximal physical capacity measured on ergometer bike, Figure 1. Patient characteristics, medication, risk factors and comorbidity will be registered. Before the exercise intervention, all participants will complete a nurse-led rehabilitation program regarding AF. The patients will then be randomized to either low intensity physical exercise (50% of maximum physical exertion) or high intensity physical exercise (75-85% of maximum physical exertion).

The exercise intervention lasts 12 weeks with exercise sessions twice weekly of 60 and 90 minutes duration, respectively. They consist of cardiovascular exercise (cycling, running) and will be supervised by cardiac physiotherapists. Intensity is measured as perceived exertion on Borg 20 scale introduced at the initial sessions. The exercise sessions will be conducted at the hospital.

During the intervention and two months after the last exercise session, the patients upload an electrocardiogram (ECG) from a handheld ECG-device twice daily and in case of symptoms of AF.

One year after the intervention is completed the patients will be invited to a follow-up. During the follow-up time all hospital admissions are registered from the medical records.

At baseline and after the 12-week intervention, blood samples will be taken and stored in a bio bank for later analyses. A total of 90 mL. blood per participant will be drawn. The blood samples will be stored anonymously at minus 80 degrees Celsius for 10 years for future research.

The trial is expected to start in October 2012 with end of follow-up one year after the last exercise session.

## Inclusion and exclusion criteria

Inclusion criteria:

1. Male or female aged ≥ 18 years
2. AF documented on ECG
3. Written informed consent

Exclusion criteria:

1. Significant language barrier
2. Severe illness with an expected survival less than one year
3. Permanent AF
4. Signs of significant cardiac comorbidity during initial testing requiring treatment (ischemic heart disease, severe aortic stenosis).

## Assessment of outcome

Burden of AF:

By using a handheld ECG device (Zenicor®), the patients transmits an ECG twice daily and in case of symptoms of arrhythmia. An episode of AF is defined as > 10 seconds with irregular frequency and no visible p-waves on ECG. Total number of AF-episodes and time to first AF-episode is registered.

One year after last the exercise session, all hospital admissions related to AF are registered from the medical records; recurrent AF, cardiac failure, stroke, dysregulated antithrombotic treatment or medical side effects, implantation of pacemaker, and surveillance of medical treatment.

Quality of Life:

At baseline and at 12-week follow up the quality of life is assessed using the Short Form 36 (SF36) and European Heart Rhythm Association score (EHRA-score).

Patient characteristics:

Age, gender, ethnicity, BMI, comorbidity, medication, cardiac risk profile (family history, smoking, hypertension, hypercholesterolemia, diabetes, stroke/TCI, claudicatio intermittens, daily physical activity, blood pressure and heart rate.

Physical capacity:

Physical capacity is measured on Six Minute Walk Test and assessment of efficiency on ergometer bike with continuously monitoring of blood pressure and ECG. A ST-depression on ECG is considered significant if it is depressed by 0.1 mV measured 60 ms after the QRS-complex. The ergometer test is considered complete when reaching 85% of maximal expected heart rate.

Cardiac load:

Blood pressure and heart rate are measured for 24 hours at baseline and 12-week follow up. Blood pressure will be reported as mean systolic and diastolic blood pressure during daytime and night time, respectively. Heart rate will be reported as mean, minimum and maximum heart rate, arrests, supraventricular and ventricular ectopic activity and number of AF-episodes.

Echocardiography is reported with standard estimates in accordance with national guidelines. Additionally, left atrium volume (ml/m^2^) will be assessed as well as diastolic function (e’, a’ and E/e’).

Biochemical and genetic markers:

General cardiac blood samples, d-dimer, vitamin D, pro-ANP, pro-BNP, SDF-1α, transforming growth factor (TGF)-β1, matrix metalloproteinase-9 (MMP), tissue inhibitor of matrix metalloproteinase-1 (TIMP-1), clusterin, telomere length, telomerase activity and copeptin. The genetic markers has no clinical consequence. Copeptin will be assessed at Rigshospitalet, Copenhagen, where smaller studies regarding AF currently are running. Telomere length, telomerase activity and fibrotic markers will be analysed by MD, ph.d. Nadia Landex.

# Outcomes

1. Primary outcome is number of AF-episodes during and after the exercise intervention measured on handheld ECG (Zenicor®)
2. Secondary outcomes are:
3. time to first AF-episode
4. change in maximal physical capacity measured on Six Minute Walk Test and ergometer bike
5. change in heart rate, blood pressure and cardiac load measured on echocardiography
6. change in quality of life
7. Tertiary outcomes are:
8. Number of hospital admissions with AF or complications to AF
9. Death
10. Change in biochemical markers

# Statistical analyses

Patient characteristics and baseline data will be analyzed using descriptive statistics. Parametric data will be reported as mean and standard deviation (SD), non-parametric data as median and range. Depending on the distribution, student-t-test or non-parametric tests will be used when comparing the intervention groups.

The effect of exercise will be analyzed in several ways depending on outcome variable; burden of AF and hospital admissions are analyzed by Poisson regression analysis, survival analyses (time-to-event) using Kaplan-Meier plots and hazard regression analysis. All analyses will be adjusted for common cardiac comorbidities and reported with two-sided confidence intervals. A p-value < 0.05 is considered statistically significant.

# Sample size

The aim of our trial is to study whether high intense physical exercise reduces burden of AF more than low intense physical exercise. Our primary outcome, burden of AF, is assessed as total number of ECGs with AF. We expect to reduce the burden of AF with 25%. The patients send an ECG twice daily and in case of symptoms which will sum up to 300 ECG per patient. We assume that total number of ECGs with AF in the low intense group will be 20 compared to 15 in the high intense group. The estimated power will be 94.3% for α=0.05 (two-sided 95% CI) and SD=6, which is higher than the generally accepted power of 80%.

# Side effects and risks

Drawing a blood sample can be painful and inconvenient when penetrating the skin and cause redness, local hematoma or infection. The electrodes used for 24-hour measurement of heart rate can cause inconvenience, itchiness or redness of the skin. Physical exercise can feel uncomfortably at high intensities. Testing maximal physical activity on ergometer bike is physically demanding and can induce discomfort, chest pain, dizziness or dyspnea in case of undiscovered cardiac disease. The test is conducted with continuous monitoring on ECG and observation of the patient by trained medical personal. Echocardiography is based on ultrasound waves, which is not associated with any short or long term risk. Handheld ECG-recording is not associated with any risk.

# Ethical considerations

Establishing a database with personal information and storing biological material is considered reasonable for the use in research.

Physical exercise is associated with several health benefits among healthy people and we find no evidence for a detrimental effect among patients with AF. Physical exercise can cause sore muscles or distortions of joint, which we try to prevent by thorough instruction and observation by physiotherapists. We expect that the patients will benefit by participating in this trial. Structured physical exercise, handheld recording of heart rhythm and nurse led education about AF is not part of standard care for patients with AF. This trial will contribute with important new research in a proper way regarding treatment of AF and guidance in physical exercise. We consider the health benefits of physical exercise to outweigh the potential risks.

Personal information will be handled in accordance with the national laws. Any new research based on this trial, which is not described in the above, has to be approved by The National Ethical Committee and the patients will be requested a new informed consent.

This protocol is written in accordance with the Helsinki Declaration. Informed consent has to be signed at inclusion to the study and covers both the participation in the exercise intervention as well as permission to store biological material. Participation is voluntary and the participant can withdraw consent at any time. Whether the patient participates in the trial does not influence on the treatment otherwise which is emphasized in the written information of the trial and in the consent.

The participants are protected in accordance with the Law of Health and the Law of Storing Personal Information. Personal safety, rights and wellbeing are beyond any scientific or societal interests. All results no matter outcome will be published in relevant medical journals and released to the public media.

We have no declarations or conflicting interests.

# References

1. Lloyd-Jones DM. Lifetime Risk for Development of Atrial Fibrillation: The Framingham Heart Study. Circulation. 2004;110: 1042–1046. doi:10.1161/01.CIR.0000140263.20897.42

2. Kannel WB, Benjamin EJ. Status of the Epidemiology of Atrial Fibrillation. Med Clin North Am. 2008;92: 17–40. doi:10.1016/j.mcna.2007.09.002

3. Psaty BM, Manolio TA, Kuller LH, Kronmal RA, Cushman M, Fried LP, et al. Incidence of and risk factors for atrial fibrillation in older adults. Circulation. 1997;96: 2455–2461.

4. Petty GW, Brown RD, Whisnant JP, Sicks JD, O’Fallon WM, Wiebers DO. Ischemic Stroke Subtypes : A Population-Based Study of Functional Outcome, Survival, and Recurrence. Stroke. 2000;31: 1062–1068. doi:10.1161/01.STR.31.5.1062

5. Benjamin EJ, Wolf PA, D’Agostino RB, Silbershatz H, Kannel WB, Levy D. Impact of Atrial Fibrillation on the Risk of Death The Framingham Heart Study. Circulation. 1998;98: 946–952. doi:10.1161/01.CIR.98.10.946

6. Reinhold T, Lindig C, Willich SN, Bruggenjurgen B. The costs of atrial fibrillation in patients with cardiovascular comorbidities--a longitudinal analysis of German health insurance data. Europace. 2011;13: 1275–1280. doi:10.1093/europace/eur116

7. Schmidt M, Jacobsen JB, Lash TL, Bøtker HE, Sørensen HT. 25 year trends in first time hospitalisation for acute myocardial infarction, subsequent short and long term mortality, and the prognostic impact of sex and comorbidity: a Danish nationwide cohort study. BMJ. 2012;344: e356.

8. Rapport om fysisk aktivitet Statens Institut for Folkesundhed.pdf.

9. Dorian P, Jung W, Newman D, Paquette M, Wood K, Ayers GM, et al. The impairment of health-related quality of life in patients with intermittent atrial fibrillation: implications for the assessment of investigational therapy. J Am Coll Cardiol. 2000;36: 1303–1309. doi:10.1016/S0735-1097(00)00886-X

10. Connolly SJ, Camm AJ, Halperin JL, Joyner C, Alings M, Amerena J, et al. Dronedarone in high-risk permanent atrial fibrillation. N Engl J Med. 2011;365: 2268–2276.

11. Chierchia GB, Capulzini L, Droogmans S, Sorgente A, Sarkozy A, Muller-Burri A, et al. Pericardial effusion in atrial fibrillation ablation: a comparison between cryoballoon and radiofrequency pulmonary vein isolation. Europace. 2010;12: 337–341. doi:10.1093/europace/eup422

12. Mont L, Elosua R, Brugada J. Endurance sport practice as a risk factor for atrial fibrillation and atrial flutter. Eur Eur Pacing Arrhythm Card Electrophysiol J Work Groups Card Pacing Arrhythm Card Cell Electrophysiol Eur Soc Cardiol. 2009;11: 11–17. doi:10.1093/europace/eun289

13. Mont L, Tamborero D, Elosua R, Molina I, Coll-Vinent B, Sitges M, et al. Physical activity, height, and left atrial size are independent risk factors for lone atrial fibrillation in middle-aged healthy individuals. Europace. 2008;10: 15–20. doi:10.1093/europace/eum263

14. Blair SN, Kohl HW, Barlow CE, Paffenbarger RS, Gibbons LW, Macera CA. Changes in physical fitness and all-cause mortality. A prospective study of healthy and unhealthy men. JAMA. 1995;273: 1093–1098.

15. Powell KE, Thompson PD, Caspersen CJ, Kendrick JS. Physical activity and the incidence of coronary heart disease. Annu Rev Public Health. 1987;8: 253–287. doi:10.1146/annurev.pu.08.050187.001345

16. Schnohr P, Lange P, Scharling H, Skov Jensen J. Long-term physical activity in leisure time and mortality from coronary heart disease, stroke, respiratory diseases, and cancer. The Copenhagen City Heart Study: Eur J Cardiovasc Prev Rehabil. 2006;13: 173–179. doi:10.1097/01.hjr.0000198923.80555.b7

17. Schnohr P, Parner J, Lange P. [Joggers live longer. The Osterbro study]. Ugeskr Laeger. 2001;163: 2633–2635.

18. Morris JN, Everitt MG, Pollard R, Chave SP, Semmence AM. Vigorous exercise in leisure-time: protection against coronary heart disease. Lancet Lond Engl. 1980;2: 1207–1210.

19. Thompson PD, Buchner D, Pina IL, Balady GJ, Williams MA, Marcus BH, et al. Exercise and physical activity in the prevention and treatment of atherosclerotic cardiovascular disease: a statement from the Council on Clinical Cardiology (Subcommittee on Exercise, Rehabilitation, and Prevention) and the Council on Nutrition, Physical Activity, and Metabolism (Subcommittee on Physical Activity). Circulation. 2003;107: 3109–3116. doi:10.1161/01.CIR.0000075572.40158.77

20. Osbak PS, Mourier M, Kjaer A, Henriksen JH, Kofoed KF, Jensen GB. A randomized study of the effects of exercise training on patients with atrial fibrillation. Am Heart J. 2011;162: 1080–1087. doi:10.1016/j.ahj.2011.09.013

21. Hegbom F, Sire S, Heldal M, Orning OM, Stavem K, Gjesdal K. Short-term exercise training in patients with chronic atrial fibrillation: effects on exercise capacity, AV conduction, and quality of life. J Cardiopulm Rehabil Prev. 2006;26: 24–29.

22. Kallergis EM, Manios EG, Kanoupakis EM, Mavrakis HE, Arfanakis DA, Maliaraki NE, et al. Extracellular matrix alterations in patients with paroxysmal and persistent atrial fibrillation: biochemical assessment of collagen type-I turnover. J Am Coll Cardiol. 2008;52: 211–215. doi:10.1016/j.jacc.2008.03.045

23. Lin C-S, Pan C-H. Regulatory mechanisms of atrial fibrotic remodeling in atrial fibrillation. Cell Mol Life Sci CMLS. 2008;65: 1489–1508. doi:10.1007/s00018-008-7408-8

24. Chu P-Y, Mariani J, Finch S, McMullen JR, Sadoshima J, Marshall T, et al. Bone marrow-derived cells contribute to fibrosis in the chronically failing heart. Am J Pathol. 2010;176: 1735–1742. doi:10.2353/ajpath.2010.090574

25. Burstein B, Nattel S. Atrial Fibrosis: Mechanisms and Clinical Relevance in Atrial Fibrillation. J Am Coll Cardiol. 2008;51: 802–809. doi:10.1016/j.jacc.2007.09.064

26. Barasch E, Gottdiener JS, Aurigemma G, Kitzman DW, Han J, Kop WJ, et al. Association between elevated fibrosis markers and heart failure in the elderly: the cardiovascular health study. Circ Heart Fail. 2009;2: 303–310. doi:10.1161/CIRCHEARTFAILURE.108.828343
