# Supplementary material for: Cardiovascular exercise and burden of arrhythmia in patients with atrial fibrillation - A randomized controlled trial
Source: PLoS One. 2017 Feb 23;12(2):e0170060. doi: 10.1371/journal.pone.0170060 (PMC5322948; doi:10.1371/journal.pone.0170060)
Supplement: S1 Clinical Trial Protocol Original Danish Version — (DOCX) [file pone.0170060.s005.docx]

**PROJEKTBESKRIVELSE**

**Fysisk træning ved atrieflimren -**

**et multidisciplinært interventionsstudie**

**Introduktion**

Forkammerflimren eller atrieflimren (AF) med uregelmæssig hjerterytme er den nye hjertemedicinske epidemi i hele den vestlige verden[1–3].

Det er en dødelig hjertesygdom, især på grund af den frygtede komplikation slagtilfælde med blodprop til hjernen, hvor 70 % af patienterne dør eller efterlades med nedsat førlighed eller et andet handicap[4,5]. Det har voldsomme følger for hver enkelt patient og udgør en stor sundhedsøkonomisk belastning med månedlange indlæggelser, efterfølgende genoptræning, nedsat erhvervsevne eller behov for øget hjælp i dagligdagen[6].

Mens andre hjertesygdomme som blodprop i hjertet nu ses sjældnere, stiger forekomsten af AF nu så markant, at hver fjerde 40-årige i dag antages at udvikle sygdommen, og antallet af patienter med AF i Danmark vil næsten fordobles inden 2020 til omkring 108.000[7,8] Årsagerne er primært forbedret behandling af de andre store hjertesygdomme som blodprop i hjertet og generelt forlænget levealder, idet AF er en sygdom, der hyppigst rammer ældre.

Udover høj risiko for indlæggelse og alvorlige komplikationer er patienterne generet af en række symptomer i dagligdagen, angst for sygdommen og for at træne på grund af hjertesygdommen samt øget risiko for udvikling af hjertesvigt på grund af nedsat pumpefunktion. Alt i alt fører dette til nedsat livskvalitet[9].

Medicinsk behandling af sygdommen er kompliceret på grund af bivirkninger og risici. Alene i 2011 er tre studier med afprøvning af hjerterytmemedicin mod AF afbrudt, fordi medicinen var forbundet med øget risiko for hjertekarsygdomme og død end den ikke-aktive behandling; heraf et meget stort internationalt studie med allerede godkendt hjerterytmemedicin[10]. Medicinsk behandling kræver derfor stadig nøje individuel vurdering af patienten og ofte indlæggelse til overvågning på grund af risiko for potentielt livsfarlige hjerterytmeforstyrrelser. Radiofrekvensablation med kateter tilbydes stadig kun et fåtal af patienterne og er forbundet med risiko for komplikationer[11].

Overordnet har vi kun begrænset viden om effekten af fysisk træning ved AF. Ekstrem og hård fysisk træning kan øge risikoen for udvikling af AF med årene[12,13], primært på grund af kammerdilatation, men motion i moderat mængde beskytter omvendt mod tidlig død og de livsstilssygdomme, der øger risikoen for AF (kranspulsåresygdom, overvægt, højt blodtryk) [14–19]. Effekten af fysisk træning på sygdommens tidlige faser kendes ikke, og mange patienter er bange for at træne med frygt for nye anfald med uregelmæssig hjerterytme eller endda hjertestop.

Træningsstudier fra vores og fra en norsk projektgruppe hos patienter med permanent AF, det vil sige med accepteret AF uden yderligere forsøg på at genoprette normal hjerterytme, viste, at træning havde gavnlig effekt på maksimal arbejdskapacitet og patienternes livskvalitet[20,21]. Nu tages disse erfaringer videre ind i næste fase: fysisk træning i et tidligere stadie af sygdommen.

Viden om patofysiologiske mekanismer ved AF er også begrænset. Fra vævsprøve-studier er der fundet tegn på kardiel fibrose (bindevævsdannelse) ved fremskreden AF-sygdom. Imidlertid er det vanskeligt at gennemføre større studier baseret på invasive undersøgelser for at udhente vævsprøver fra hjertet, men det er muligt i blodprøver at påvise fibrosemarkører, der afspejler fibrose i atrierne[22–24]. Vi har derfor udført pilotstudier med blodprøver og kan påvise signifikant association mellem fibrosemarkører og AF, hvilket bekræfter tidligere rapporter om sammenhængen mellem atriefibrose og udvikling af AF.

Vi fandt således, at stromal derived factor (SDF)-1α, der rekrutterer stamceller fra knoglemarven til hjertet og stimulerer angiogenese, var signifikant øget hos patienter med permanent AF sammenlignet med patienter med paroxystisk (AF-anfald med spontan konvertering til sinusrytme) AF efter korrektion for andre forklarende variable inklusiv alder. Ligeledes så vi, at koncentrationen af clusterin, der anses som markør for biologisk aldring, var højere hos AF-patienter end kontrolniveau hos raske. Aktuelt undersøger vi i et samarbejde med Rigshospitalet proteinet copeptin, der er vist at være relateret til hjertesvigt.

Øget bindevævsdannelse i hjertets forkamre, ledsagende hjertesvigt og tegn på biologisk aldring kan have betydning for prognosen, især i forhold til udvikling af permanent AF, udvikling af de alvorlige komplikationer slagtilfælde, hjertesvigt og død[25,26].

I det her beskrevne studie vil vi desuden undersøge specifikke genetiske markører, såkaldt telomerlængde og telomerase-aktivitet. Telomerer er de ikke-kodende DNA-sekvenser i enderne af kromosomerne. De forkortes med alderen, og med øget hastighed ved biologisk/ psykisk stress, og når de når en kritisk længde, mister cellerne evnen til at dele sig og flere andre specialiserede funktioner.

Imidlertid er telomerlængden dynamisk; enzymet telomerase kan forlænge telomererne, hvilket kan udskyde det tidspunkt, hvor cellen mister sine funktioner. Kort telomerlængde er rapporteret associeret til kranspulsåresygdom og hjertesvigt; man har ingen viden om betydning ved AF. Enkelte forsøg har vist sammenhæng mellem fysisk aktivitet og telomerlængde, måske gennem en øget aktivitet af telomerase, men der savnes præcise data på dette område. Vi ønsker at undersøge, om kort telomerlængde er associeret til grad af og prognose ved AF, og om træning øger telomerase-aktivitet og telomerlængde, samt hvorvidt disse markører hos den enkelte patient prædikterer respons på træning.

Sygdommen AF optræder i mange kliniske sammenhænge, og behandlingen differentieres afhængig af disse. Vi planlægger derfor også at vurdere flere effektmål i dette interventionsstudie. Projektgruppen besidder kompetencer til at angribe sygdommen på denne måde. Vi vil måle effekt på risiko for AF-recidiv, effekt på livskvalitet, effekt på risiko for indlæggelse og AF-komplikationer, effekt på patienternes fysiske arbejdskapacitet, på hjertets belastningsgrad og biokemiske markører for belastning af hjertet og bindevævsdannelse (fibrose) i atrierne.

Der planlægges derfor et interventionsstudie af konsekutivt inkluderede patienter med EKG-dokumenteret paroxystisk eller persisterende AF randomiseret til let eller moderat-hård fysisk træning.

Ved et design med både let og moderat-hård fysisk træning undgås, at en træningsfri kontrolgruppe selv-træner uden vores viden; i stedet fungerer gruppen med let træning som kontrol. Vi kan samtidig undersøge, om der er en slags tærskelværdi for gavnlig effekt af træning og en eventuel advarsel om for hård træning.

Hvis fysisk træning kan anvendes som nyt supplement til de kendte behandlinger og dermed forbedre prognose og livskvalitet hos denne store patientpopulation, er perspektiverne store med en ny behandlingsstrategi ved hjerterytmeforstyrrelser.

**Studiets formål er således at undersøge**

1. effekt af fysisk træning på risiko for anfald af AF bedømt på hjemme-EKG (Zenicor®) under og efter træningsperioden og på risiko for indlæggelser, alvorlige komplikationer eller død registreret over et år
2. effekt af fysisk træning over 12 uger målt på patienternes livskvalitet (SF36) og EHRA-klassifikation henholdsvis før og efter træningsperioden samt efter ét år
3. effekt af fysisk træning på maksimal arbejdskapacitet hos patienter med AF (gangtest, arbejds-EKG)
4. effekt af fysisk træning på hjertets belastning vurderet ved avanceret ekkokardiografi, hjerterytmen målt over et døgn, døgnblodtryk og blodprøver
5. telomerlængde og telomeraseaktivitet hos AF-patienter og om disse genetiske markører prædikterer effekt af træning og om de ændres (bedres) af træning
6. koncentration af en række fibrosemarkører og proteinet copeptin hos patienter med paroxystisk AF og hvorvidt niveauerne af disse markører ændres ved fysisk træning, samt om niveauet af fibrosemarkører og copeptin ved studiets start prædikterer effekt af træning

**Studiets hypoteser er at**

1. moderat-hård fysisk træning medfører en reduktion af AF-byrde og AF-relaterede indlæggelser (tid til AF-anfald, antal AF-anfald og indlæggelsesdage over et år) på minimum 20 %
2. moderat-hård fysisk træning medfører 20 % forbedret livskvalitet på minimum fem af otte parametre i SF36-skalaen og EHRA-klassifikation
3. moderat-hård fysisk træning medfører en øgning af maksimal arbejdskapacitet på minimum 20 %
4. moderat-hård fysisk træning medfører signifikant lavere gennemsnitlig puls og blodtryk målt over et døgn samt reduceret venstre ventrikel diastole-funktion målt på vævs-doppler-ekkokardiografi. Ingen signifikante ændringer målt ved konventionel ekkokardiografi. Der prædikteres ingen ændringer i almindelige rutineblodprøver, hs-CRP eller natriuretiske peptider (proANP og proBNP)
5. der ses en signifikant association mellem telomerlængde og respons på fysisk træning (målt på punkt 1,3 og 4) samt øgning af telomeraseaktivitet efter træningsperioden
6. Udgangsniveau for både fibrosemarkører og copeptin er signifikant øget sammenlignet med oplyste referenceområdet hos kontrolgrupper samt associeret til samlet antal indlæggelser og AF-byrde i løbet af opfølgningsperioden. Koncentrationen af copeptin reduceres signifikant ved moderat-hård fysisk træning. Ingen ændring i fibrose-markører

**Metode – design**

Studiet er designet som et randomiseret, enkeltblindet interventionsstudie. Observatører der udfører måling af effektmål er blindet i forhold til randomiseringen.

Patienterne randomiseres ved almindelig lodtrækning. Af hensyn til træningsplanlægning og holdstørrelse fordeles patienterne i tre blokke, hver med to grupper på 10 patienter. I 20 konvolutter lægges henholdsvis bogstav A eller B fordelt ligeligt. Bogstavet symboliserer træningsgruppen, A trækning betyder moderat-hård træning. Konvolutterne samles i en større konvolut, hvorfra patienten trækker tilfældigt.

Der undersøges hos 60 patienter med anfald af AF effekt af træning - let fysisk træning versus moderat-hård fysisk træning. Patienterne inkluderes løbende ved personlig henvendelse fra Hvidovre Hospitals Kardiologiske Afdeling fra sengeafsnit, ambulatorier og den Akutte Modtageafdeling.


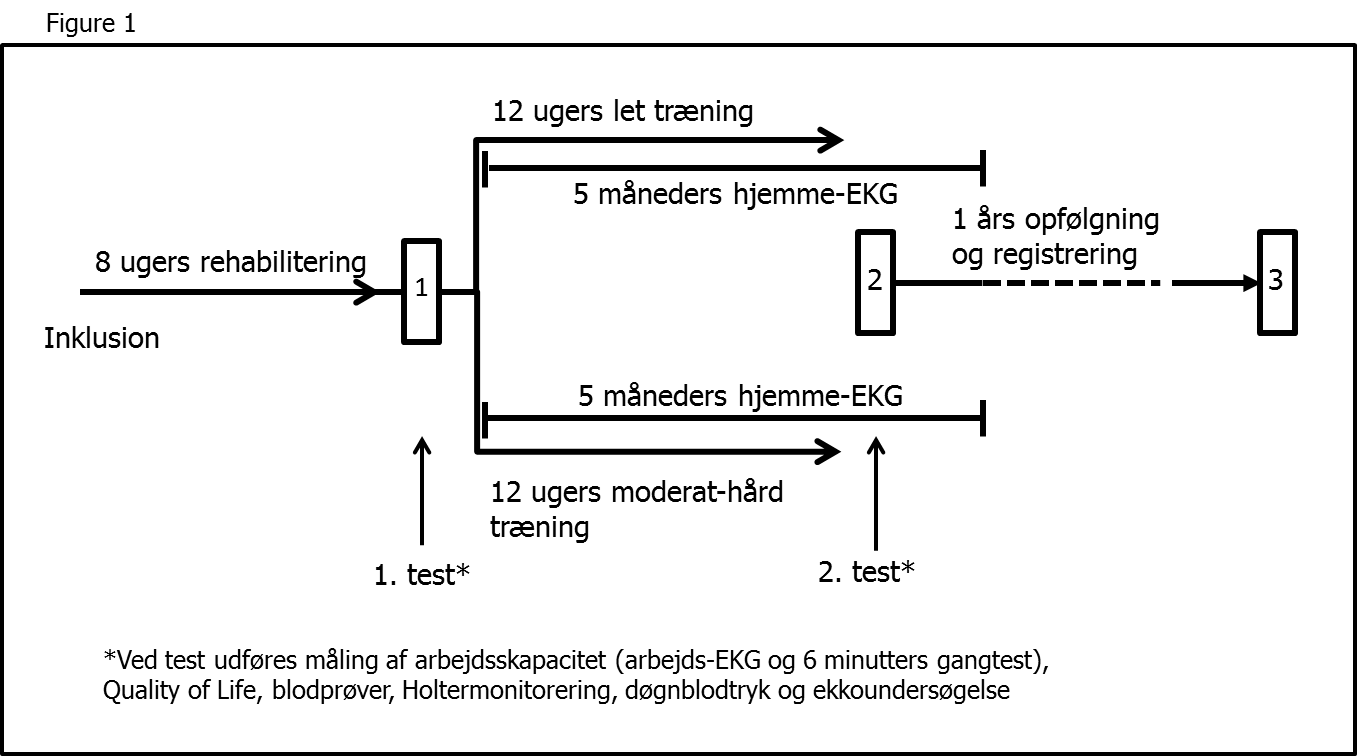


Efter inklusion udføres indledende tests (se figur 1) herunder måling af arbejdskapacitet med standardiseret seks minutters gangtest og måling af maksimal iltoptagelse ved ergometertest. Indledende og opfølgende tests udføres af blinded personale udenfor forskningsgruppen og foretages med minimum 2 dages restitution i mellem. Patienternes karakteristika, medicin, risikofaktorer og comorbiditet registreres.

Begge grupper får først et otte uger langt forløb med konsultationer hos hjertesygeplejersker (rehabilitering) for at sikre viden om sygdommen og forståelse af den medicinske behandling, samt vanligt individuelt behandlingsforløb. Dermed sikres, at vi udelukkende måler effekt af træning. Herefter randomiseres patienterne til de to træningsgrupper let og moderat-svær belastning (50% og 75-85 % af maksimal belastning).

Træningsperioden planlægges med i alt seks hold á 10 patienter fordelt på tre 12-ugers-perioder. Den består af holdtræning 2 gange ugentligt hhv. 60 + 90 minutter under vejledning af en hjertefysioterapeut Malene Mourier. Træningsintensitet tilrettelægges individuelt ud fra indledende tests og vurderes under træningen med pulsmåling og egen opfattelse af intensitet med Borgskala. Der laves udelukkende kredsløbstræning (løb, cykling, trapper) med opvarmning og nedkøling. Træningssessionerne vil foregå i hospitalets træningslokaler.

Under træningsperioden og to måneder efter endt træning foretages hjemmemålinger af hjerterytmen med håndholdt apparat fra Zenicor. Patienten instrueres i to gange dagligt samt ved symptomer på AF at indsende et hjertediagram (EKG).

Et år efter afsluttet træning indkaldes de til opfølgende undersøgelser, se figur 1.

Projektet vil starte oktober 2012, andet og tredje træningshold starter henholdsvis februar og august 2013. Opfølgning afsluttes et år efter sidste holdstart det vil sige august 2014. I den ét år lange opfølgningsperiode registreres alle AF-relaterede indlæggelser ved søgning i patientjournaler.

Ved inklusion og de efterfølgende to test tages blodprøver til biobank med henblik på måling af biokemiske og genetiske markører beskrevet nedenfor i målparametre. Der udtages 30 mL pr. gang det vil sige i alt 90 mL. pr. deltager. Vi anvender i alt 8 glas; 1 citratglas, 3 heparinglas, 1 EDTA-trasylolglas, 2 tørglas og 1 EDTA-glas.

Blodprøverne er anonymiseret og opbevares i afdelingens forskningsfryser ved -80 grader. De bliver opbevaret i 10 år efter forsøgets afslutning til evt. videre forskning. Forsøgsdeltagerne kan efter ønske få deres blodprøver destrueret efter forsøget.

##### Metode: patientpopulation – in- og eksklusionskriterier

Inklusionskriterier til studiet:

1. mænd og kvinder med alder over 18 år
2. EKG-dokumenteret anfald af AF
3. informeret mundtligt og skriftligt samtykke.

Eksklusionskriterier:

1. patienter med manglende dansk-kendskab i en grad, der forhindrer deltagelse
2. patienter med alvorlig sygdom med forventet overlevelse kortere end et år eller helbredsforhold, der forhindrer træning.
3. etableret og accepteret permanent AF.
4. hvis der ved indledende undersøgelser fremkommer tegn på betydende hjertesygdom (iskæmisk hjertesygdom, svær aortastenose) som kræver umiddelbar intervention.

**Metode - måleparametre**

**AF-byrde:**

Der optages med et nyudviklede telemedicinske udstyr hjemme-EKG’er, der indsendes morgen, aften samt ved symptomer på arytmi fra start på træningsperioden til 2 måneder efter afsluttet træning, det vil sige i alt 5 måneder.

Der registreres antal anfald og tid til anfald. Et AF-anfald defineres som periode på EKG > 10 sekunder uden synlige p-takker og med uregelmæssig ventrikelfrekvens.

I den ét år lange opfølgningsperiode fra start på træningsperiode og et år efter afsluttet træningsperiode registreres alle indlæggelser samt varighed af indlæggelser relateret til AF: recidiv AF, hjertesvigt, apopleksia cerebri, dysreguleret AK-behandling, andre medicinbivirkninger, pacemakerimplantation, elektiv indlæggelse til medicinregulering. Der søges i patientjournaler og Grønt System.

**Livskvalitet:**

Der måles før rehabilitering, før træning, efter træning og ved afsluttet follow-up periode livskvalitet på SF36 samt den nyudviklede EHRA-skala.

**Klinisk registrering:**

Der registreres alder, køn, etniske forhold, co-morbiditet, medicin, kardial risikoprofil (arv, rygning, hypertension, hypercholesterolæmi, diabetes, TCI/apopleksi/claudicatio), BMI, fysisk aktivitet, BT, puls, saturation, kliniske fund, blodprøvesvar, resultaterne af de parakliniske undersøgelser.

**Maksimal arbejdskapacitet:**

Vurderes før og efter træningsperiode samt efter et år ved seks-minutters gangtest og arbejds-ekg med supplerende beregning af effektivitet. Arbejds-EKG foregår på ergometercykel med kontinuerlig 12 aflednings-EKG monitorering og løbende blodtrykskontrol. Signifikant ST-depression er horisontal eller descenderende ST-depression på mindst 0,1 mV målt 60 ms efter QRS komplekset. Målingerne udføres på tre konsekutive og repræsentative komplekser. Testen klassificeres som nondiagnostisk, hvis hjertefrekvensen ikke når 85 % af den maksimale hjertefrekvens (under hensyntagen til alder og køn).

**Kardiel belastning af AF:**

Før og efter træningsperiode samt efter et år gennemføres et døgns måling af blodtryk med angivelse af middelblodtryk om dagen og om natten samt pulstryk og måling af hjerterytme (hjemme-Holtermonitorering) med angivelse af middelhjertefrekvens, maksimal og minimal hjertefrekvens, pauser, supraventrikulær og ventrikulær ektopisk aktivitet samt antal og varighed af AF-episoder.

Ekkokardiografi udføres på samme tidspunkter som en standard transthorakal ekkokardiografi. Der angives alle standardmål for ekkokardiografi efter Dansk Cardiologisk Selskabs retningslinjer suppleret med volumenmålinger af venstre atrium (ml/m2), vævs-Doppler ekkokardiografi med estimering af e’, a’ og a’ og deraf deriveret E/e’.

**Biokemiske og genetiske markører:**

Der foretages rutineprøver, d-dimer, D-vitamin, pro-ANP, pro-BNP samt måles indholdet af stromal derived factor (SDF)-1α og transforming growth factor (TGF)-β1, enzymerne matrix metalloproteinase-9 (MMP) og tissue inhibitor of matrix metalloproteinase-1 (TIMP-1), clusterin, telomerlængde og telomeraseaktivitet samt copeptin. Resultater fra de genetiske prøver har ingen behandlingskonsekvens.

Copeptin måles hos overlæge Jens Peter Götze, Klinisk Biokemisk Afdeling, Rigshospitalet, hvor der aktuelt køres pilotstudier på en mindre AF-population.

Telomerlængde og telomerase-aktivitet måles sammen med de beskrevne fibrosemarkører i et samarbejde mellem læge, ph.d. Nadia Landex, Hjertemedicinsk Afdeling og Klinisk Biokemisk Afdeling på Hvidovre Hospital under ledelse af overlæge Jørgen Hjelm Poulsen. Nadia Landex har siden januar 2012 optimeret teknikker og arbejdsinstrukser til måling af fibrosemarkører og vist at analyserne kan gennemføres med tilstrækkelig præcision og reproducerbarhed.

#### Prioriterede effektmål er således:

1. Primære effektmål er antal af AF-anfald under og efter træningsperioden målt med hjemme-EKG-optagelse (Zenicor).
2. Sekundære effektmål er
   1. tid til første AF-anfald.
   2. ændring af livskvalitet hhv. efter hjerterehabilitering og efter træningsperioden
   3. ændring af maximal fysisk arbejdskapacitet bedømt ved arbejds-EKG og 6 minutters gangtest.
   4. Ændring af ekkokardiografiske mål, døgnblodtryk og hjerterytme ved Holtermåling
3. Tertiære effektmål er
   1. Antal indlæggelser med anfaldsvis AF, komplikationer til AF.
   2. Død
   3. Ændring i biokemiske markører

###### Metode - statistik

Deskriptiv statistik ved analyse af patientkarakteristika og parakliniske baseline-værdier.

Ved normalfordeling af data rapporteres middelværdi og spredning; ved skæv fordeling angives median med rækkevidde. I det omfang, de statistiske modeller kræver, vil ikke-normalfordelte data blive transformeret til normalfordelte.

I sammenligning med de to patientgrupper anvendes ved normalfordelte data parret student t-test; i modsat fald non-parametriske test.

I analyse af træningseffekt udføres flere separate analyser med henholdsvis antal registrerede AF-episoder, tid til første AF-episode, antal indlæggelser og antal indlæggelsesdage som effektvariable: Der anvendes logistisk multipel regressionsanalyse for effektvariablen indlæggelse versus ingen indlæggelse, Poisson regressionsanalyse til analyse af højt antal versus få hændelser (anfald, indlæggelser, indlæggelsesdage), samt overlevelses-analyser (tid-til-event) med Kaplan-Meier plots og hazard regressionsanalyse med udregning af log-rank-estimater.

I alle effektanalyser analyser korrigeres for hyppigste ledsagesygdomme (taget fra CHA_2_DS_2_-VASc-scoren) som forklarende variable. Der anvendes tosidede sikkerheds-intervaller, da vi på forhånd ikke kan udelukke en forværring af AF ved træning.

Ændring i måleparametre over tid, det vil sige før og efter træning samt ved langtidsopfølgning, analyseres ved ANOVA-analyser med patienterne som egne kontroller.

Generelt tolkes P-værdier lavere end 0,05 som værende signifikante.

**Styrkeberegning**

Vi ønsker at undersøge, hvorvidt moderat-hård fysisk træning sammenlignet med let træning reducerer AF-byrden. Effektmålet defineres her som antal EKG-målinger med AF. Styrkeberegning på en reduktion med 25 % bygger på følgende antagelser: Monitoreres patienterne med EKG-hjemmemålinger morgen og aften i træningsperioden og de efterfølgende to måneder samt ved symptomer fås i alt mere end 300 målinger per patient i løbet af de fem måneder. Sættes α = 0,05 svarende til 95 % sikkerhedsintervaller (tosidede) og antages antal målinger med AF i kontrolgruppen - konservativt vurderet - at udgøre i antal 20 mod 15 i gruppen med moderat-hård træning, det vil sige 25 % lavere, med standard deviation sat til 6, fås en styrke på 94,3 %, hvilket er sikkert højere end den vanligt accepterede styrke på 80 %.

**Projektgruppens kompetencer og muligheder for at gennemføre studiet**

Undertegnede læge Ane Katrine Skielboe indskrives som ph.d.-studerende ved Københavns Universitet 1. oktober 2012.

Vi har opbygget et tæt, tværfagligt netværk og samarbejde med førende hjerteafdelinger i ind- og udland (Rigshospitalet, Gentofte Hospital og The University of Rochester Medical Center, New York i USA). Der er en stab af faste medarbejdere med spidskompetencer på hver deres felt: projektsygeplejersker, bioanalytikere, en hjertefysioterapeut med erfaring med træning af patienter med AF og projektsekretærer. Holterlaboratoriet har – efter vor viden som det eneste - ISO-certificering fra Dansk Standard. Ulrik Dixen er afsnitsansvarlig overlæge ved Hjerteafdelingen på Hvidovre Hospital og klinisk lektor (A-lektor) ved Københavns Universitet. Ulrik Dixen har vejledt mere end 35 medicinstuderende med opgaver og artikler, har vejledt ved og bedømt ph.d.-afhandlinger samt undervist og eksamineret medicinstuderende.

**Bivirkninger, risisci og ulemper**

Ved blodprøvetagning er der risiko for ubehag eller smerte i forbindelse med perforation af huden, rødmen, irritation eller lokalt hæmatom samt lokal hudinfektion.

Ved Holtermonitorering kan der opstå let ubehag, kløe og rødmen hvor elektroder placeres.

Fysisk træning kan medføre almindeligt ubehag relateret til hårdt fysisk arbejde.

Arbejds-EKG er fysisk belastende og kan ved uerkendt hjertesygdom udløse utilpashed, brystsmerter, svimmelhed, besvimelser eller åndenød. Den foretages under konstant EKG-monitorering og tæt observation af læge og sygeplejerske.

Ekkoundersøgelse er baseret på ultralydsbølger og ikke forbundet med korttids –eller langtidsrisici.

Hjemme-EKG er ikke forbundet med risiko eller ulempe.

### Videnskabsetisk redegørelse

Etablering af en forskningsbiobank og en database giver anledning til videnskabsetiske overvejelser, da forsøgspersonernes biologiske materiale nedfryses med henblik på senere forskning, og da der registreres personfølsomme informationer om forsøgspersonerne. Den potentielle risiko, dette udgør, vurderes ikke at have et uforsvarligt omfang.

Fysisk træning er ved adskillige undersøgelser vist at være forbundet med betydelige helbredsmæssige fordele hos raske personer og der findes ikke belæg for, at det skulle være skadeligt hos den valgte population. Ved træning kan der opstå muskelømhed, overbelastningsskader eller forvridning af led. Dette imødekommes ved grundig individuel instruktion og observation af en fysioterapeut.

Det forventes, at forsøgsdeltagerne opnår personlig nytte ved at deltage i forsøget. Fysisk træning, hjemmemålinger af hjerterytmen med det nye håndholdte apparatur og et planlagt forløb med konsultationer hos hjertesygeplejersker er alle nye tiltag, der ikke indgår i afdelingens vanlige behandling af denne sygdom.

Vi anser derfor denne undersøgelse for på forsvarlig vis at bringe væsentlig ny viden indenfor behandling af en hastigt voksende patientgruppe, der kan være kompliceret at behandle medicinsk eller med ablation. Vi vil fremover på et stærkere grundlag kunne vejlede patienter med AF om gavnlig effekt eller eventuelle begrænsninger ved fysisk udfoldelse.

Således kan studiet bane vejen til behandling af en folkesygdom inden for hjertemedicin og give os muligheder for at forbedre og individualisere behandlingen.

Den sundhedsmæssige gevinst, der kan opnås for fremtidige patienter gennem forskningsprojektet, vurderes at opveje den potentielle risiko.

Oplysninger om forsøgspersonerne beskyttes efter lov om behandling af personoplysninger og sundhedsloven.

Videnskabsetisk komité skal godkende et nyt forskningsprojekt, når det bliver aktuelt at bruge prøverne til formål udover de beskrevne, og som udgangspunkt skal der gives nyt samtykke fra forsøgspersonen.

Protokollen er udarbejdet i henhold til Helsinki-deklarationen. Skriftligt informeret patientsamtykke skal foreligge før inklusion i studiet og omfatter informeret samtykke til deltagelse i forskningsprojektet vedrørende etablering af database samt opbevaring af deltagerens biologiske materiale i en forskningsbiobank.

Deltagelse i studiet er frivillig, og deltageren kan på et hvilket som helst tidspunkt trække sit samtykke tilbage. Hvorvidt patienten deltager eller ej får ingen konsekvenser for den daglige behandling på afdelingen, hvilket understreges i den skriftlige patientinformation og samtykkeerklæringen.

**Respekten for forsøgspersonernes fysiske og mentale integritet samt privatlivets fred.**

Forsøgspersonerne beskyttes efter loven om behandling af personoplysninger og sundhedsloven.

Hensynet til forsøgspersonens sikkerhed, rettigheder og velbefindende går forud for videnskabelige og samfundsmæssige interesser.

Projektet anmeldes til Datatilsynet. Oplysninger fra patientjournalerne relateret til patienternes atrieflimren og til den kardielle risikoprofil registreres.

Alle resultater uanset udfald vil blive publiceret i lægevidenskabelige tidsskrifter og formidlet i offentlige relevante medier.

**Budget og finansiering**

Projektet er opstartet på initiativ af overlæge, klinisk ekstern lektor, ph.d. Ulrik Dixen.

Der henvises venligst til detaljeret budget. Der er opnået støtte fra flere fonde på i alt 635.000 kroner. Dette beløb dækker løn til projektansat personale i nøglefunktioner (fysioterapeut til træning, sygeplejerske til rehabilitering og en projektsekretær). Desuden er der opnået støtte til leje af Zenicor EKG-hjemmemålere og fuld finansiering af forskningsfryser til opbevaring af de beskrevne blodprøver på minus 80 grader.

Der søges fra fonde til finansiering af projektansvarlige læges løn, og afdelingens forskningsfond vil supplere med manglende midler. Der foreligger ingen økonomiske interessekonflikter.

Kontaktperson:

Ane Katrine Skielboe,

Hvidovre Hospital, Medicinsk Enhed, Kardiologisk Afdeling,

Kettegård Allé 30,

2650 Hvidovre

**Referencer**

1. Lloyd-Jones DM. Lifetime Risk for Development of Atrial Fibrillation: The Framingham Heart Study. Circulation. 2004;110: 1042–1046. doi:10.1161/01.CIR.0000140263.20897.42

2. Kannel WB, Benjamin EJ. Status of the Epidemiology of Atrial Fibrillation. Med Clin North Am. 2008;92: 17–40. doi:10.1016/j.mcna.2007.09.002

3. Psaty BM, Manolio TA, Kuller LH, Kronmal RA, Cushman M, Fried LP, et al. Incidence of and risk factors for atrial fibrillation in older adults. Circulation. 1997;96: 2455–2461.

4. Petty GW, Brown RD, Whisnant JP, Sicks JD, O’Fallon WM, Wiebers DO. Ischemic Stroke Subtypes : A Population-Based Study of Functional Outcome, Survival, and Recurrence. Stroke. 2000;31: 1062–1068. doi:10.1161/01.STR.31.5.1062

5. Benjamin EJ, Wolf PA, D’Agostino RB, Silbershatz H, Kannel WB, Levy D. Impact of Atrial Fibrillation on the Risk of Death The Framingham Heart Study. Circulation. 1998;98: 946–952. doi:10.1161/01.CIR.98.10.946

6. Reinhold T, Lindig C, Willich SN, Bruggenjurgen B. The costs of atrial fibrillation in patients with cardiovascular comorbidities--a longitudinal analysis of German health insurance data. Europace. 2011;13: 1275–1280. doi:10.1093/europace/eur116

7. Schmidt M, Jacobsen JB, Lash TL, Bøtker HE, Sørensen HT. 25 year trends in first time hospitalisation for acute myocardial infarction, subsequent short and long term mortality, and the prognostic impact of sex and comorbidity: a Danish nationwide cohort study. BMJ. 2012;344: e356.

8. Rapport om fysisk aktivitet Statens Institut for Folkesundhed.pdf.

9. Dorian P, Jung W, Newman D, Paquette M, Wood K, Ayers GM, et al. The impairment of health-related quality of life in patients with intermittent atrial fibrillation: implications for the assessment of investigational therapy. J Am Coll Cardiol. 2000;36: 1303–1309. doi:10.1016/S0735-1097(00)00886-X

10. Connolly SJ, Camm AJ, Halperin JL, Joyner C, Alings M, Amerena J, et al. Dronedarone in high-risk permanent atrial fibrillation. N Engl J Med. 2011;365: 2268–2276.

11. Chierchia GB, Capulzini L, Droogmans S, Sorgente A, Sarkozy A, Muller-Burri A, et al. Pericardial effusion in atrial fibrillation ablation: a comparison between cryoballoon and radiofrequency pulmonary vein isolation. Europace. 2010;12: 337–341. doi:10.1093/europace/eup422

12. Mont L, Elosua R, Brugada J. Endurance sport practice as a risk factor for atrial fibrillation and atrial flutter. Eur Eur Pacing Arrhythm Card Electrophysiol J Work Groups Card Pacing Arrhythm Card Cell Electrophysiol Eur Soc Cardiol. 2009;11: 11–17. doi:10.1093/europace/eun289

13. Mont L, Tamborero D, Elosua R, Molina I, Coll-Vinent B, Sitges M, et al. Physical activity, height, and left atrial size are independent risk factors for lone atrial fibrillation in middle-aged healthy individuals. Europace. 2008;10: 15–20. doi:10.1093/europace/eum263

14. Blair SN, Kohl HW, Barlow CE, Paffenbarger RS, Gibbons LW, Macera CA. Changes in physical fitness and all-cause mortality. A prospective study of healthy and unhealthy men. JAMA. 1995;273: 1093–1098.

15. Powell KE, Thompson PD, Caspersen CJ, Kendrick JS. Physical activity and the incidence of coronary heart disease. Annu Rev Public Health. 1987;8: 253–287. doi:10.1146/annurev.pu.08.050187.001345

16. Schnohr P, Lange P, Scharling H, Skov Jensen J. Long-term physical activity in leisure time and mortality from coronary heart disease, stroke, respiratory diseases, and cancer. The Copenhagen City Heart Study: Eur J Cardiovasc Prev Rehabil. 2006;13: 173–179. doi:10.1097/01.hjr.0000198923.80555.b7

17. Schnohr P, Parner J, Lange P. [Joggers live longer. The Osterbro study]. Ugeskr Laeger. 2001;163: 2633–2635.

18. Morris JN, Everitt MG, Pollard R, Chave SP, Semmence AM. Vigorous exercise in leisure-time: protection against coronary heart disease. Lancet Lond Engl. 1980;2: 1207–1210.

19. Thompson PD, Buchner D, Pina IL, Balady GJ, Williams MA, Marcus BH, et al. Exercise and physical activity in the prevention and treatment of atherosclerotic cardiovascular disease: a statement from the Council on Clinical Cardiology (Subcommittee on Exercise, Rehabilitation, and Prevention) and the Council on Nutrition, Physical Activity, and Metabolism (Subcommittee on Physical Activity). Circulation. 2003;107: 3109–3116. doi:10.1161/01.CIR.0000075572.40158.77

20. Osbak PS, Mourier M, Kjaer A, Henriksen JH, Kofoed KF, Jensen GB. A randomized study of the effects of exercise training on patients with atrial fibrillation. Am Heart J. 2011;162: 1080–1087. doi:10.1016/j.ahj.2011.09.013

21. Hegbom F, Sire S, Heldal M, Orning OM, Stavem K, Gjesdal K. Short-term exercise training in patients with chronic atrial fibrillation: effects on exercise capacity, AV conduction, and quality of life. J Cardiopulm Rehabil Prev. 2006;26: 24–29.

22. Kallergis EM, Manios EG, Kanoupakis EM, Mavrakis HE, Arfanakis DA, Maliaraki NE, et al. Extracellular matrix alterations in patients with paroxysmal and persistent atrial fibrillation: biochemical assessment of collagen type-I turnover. J Am Coll Cardiol. 2008;52: 211–215. doi:10.1016/j.jacc.2008.03.045

23. Lin C-S, Pan C-H. Regulatory mechanisms of atrial fibrotic remodeling in atrial fibrillation. Cell Mol Life Sci CMLS. 2008;65: 1489–1508. doi:10.1007/s00018-008-7408-8

24. Chu P-Y, Mariani J, Finch S, McMullen JR, Sadoshima J, Marshall T, et al. Bone marrow-derived cells contribute to fibrosis in the chronically failing heart. Am J Pathol. 2010;176: 1735–1742. doi:10.2353/ajpath.2010.090574

25. Burstein B, Nattel S. Atrial Fibrosis: Mechanisms and Clinical Relevance in Atrial Fibrillation. J Am Coll Cardiol. 2008;51: 802–809. doi:10.1016/j.jacc.2007.09.064

26. Barasch E, Gottdiener JS, Aurigemma G, Kitzman DW, Han J, Kop WJ, et al. Association between elevated fibrosis markers and heart failure in the elderly: the cardiovascular health study. Circ Heart Fail. 2009;2: 303–310. doi:10.1161/CIRCHEARTFAILURE.108.828343
